# A Case for Automated Segmentation of MRI Data in Milder Neurodegenerative Diseases

**DOI:** 10.1101/2025.02.18.25322304

**Published:** 2025-02-20

**Authors:** Connor J. Lewis, Jean M. Johnston, Precilla D’Souza, Josephine Kolstad, Christopher Zoppo, Zeynep Vardar, Anna Luisa Kühn, Ahmet Peker, Zubir S. Rentiya, William A. Gahl, Mohammed Salman Shazeeb, Cynthia J. Tifft, Maria T. Acosta

**Affiliations:** Office of the Clinical Director and Medical Genetics Branch, National Human Genome Research Institute, 10 Center Drive, Bethesda MD USA; Office of the Clinical Director and Medical Genetics Branch, National Human Genome Research Institute, 10 Center Drive, Bethesda MD USA; Office of the Clinical Director and Medical Genetics Branch, National Human Genome Research Institute, 10 Center Drive, Bethesda MD USA; McLean Hospital, Belmont, MA, USA; Department of Radiology, University of Massachusetts Chan Medical School, Worcester MA USA; Department of Radiology, University of Massachusetts Chan Medical School, Worcester MA USA; Department of Radiology, University of Massachusetts Chan Medical School, Worcester MA USA; Koç University Hospital, Istanbul, Türkiye; Department of Radiation Oncology & Radiology, University of Virginia, Charlottesville, VA, USA; Medical Genetics Branch, National Human Genome Research Institute, 10 Center Drive, Bethesda MD USA; Department of Radiology, University of Massachusetts Chan Medical School, Worcester MA USA; Office of the Clinical Director and Medical Genetics Branch, National Human Genome Research Institute, 10 Center Drive, Bethesda MD USA; Office of the Clinical Director and Medical Genetics Branch, National Human Genome Research Institute, 10 Center Drive, Bethesda MD USA

**Keywords:** GM1 Gangliosidosis, Imaging Biomarkers, MRI Segmentation

## Abstract

**Background:**

Volumetric analysis and segmentation of magnetic resonance imaging (MRI) data is an important tool for evaluating neurological disease progression and neurodevelopment. Fully automated segmentation pipelines offer faster and more reproducible results. However, since these analysis pipelines were trained on or run based on atlases consisting of neurotypical controls, it is important to evaluate how accurate these methods are for neurodegenerative diseases. In this study, we compared 5 fully automated segmentation pipelines including FSL, Freesurfer, volBrain, SPM12, and SimNIBS with a manual segmentation process in GM1 gangliosidosis patients and neurotypical controls.

**Methods:**

We analyzed 45 MRI scans from 16 juvenile GM1 gangliosidosis patients, 11 MRI scans from 8 late-infantile GM1 gangliosidosis patients, and 19 MRI scans from 11 neurotypical controls. We compared results for 7 brain structures including volumes of the total brain, bilateral thalamus, ventricles, bilateral caudate nucleus, bilateral lentiform nucleus, corpus callosum, and cerebellum.

**Results:**

We found volBrain’s *vol2Brain* pipeline to have the strongest correlations with the manual segmentation process for the whole brain, ventricles, and thalamus. We also found Freesurfer’s *recon-all* pipeline to have the strongest correlations with the manual segmentation process for the caudate nucleus. For the cerebellum, we found a combination of volBrain’s *vol2Brain* and SimNIBS’ *headreco* to have the strongest correlations depending on the cohort. For the lentiform nucleus, we found a combination of *recon-all* and FSL’s *FIRST* to give the strongest correlations depending on the cohort. Lastly, we found segmentation of the corpus callosum to be highly variable.

**Conclusion:**

Previous studies have considered automated segmentation techniques to be unreliable, particularly in neurodegenerative diseases. However, in our study we produced results comparable to those obtained with a manual segmentation process. While manual segmentation processes conducted by neuroradiologists remain the gold standard, we present evidence to the capabilities and advantages of using an automated process including the ability to segment white matter throughout the brain or analyze large datasets, which pose feasibility issues to fully manual processes. Future investigations should consider the use of artificial intelligence-based segmentation pipelines to determine their accuracy in GM1 gangliosidosis, lysosomal storage disorders, and other neurodegenerative diseases.

## Introduction

Volumetric analysis of magnetic resonance imaging (MRI) has proven useful in diagnosing and monitoring the progression of various neurological disorders.^1,2^ The capability to evaluate brain growth and development has provided important insights in brain development and pathogenesis.^3^ Numerous techniques for performing these volumetric calculations exist, including both manual and automated methods.^4,5^

Manual segmentation processing of MRI data involves a clinician manually tracing the structure of interest slice by slice. While these approaches are accurate, they are also time consuming and unrealistic for analyzing larger datasets.^6^ Inter-rater reliability issues are also noteworthy for manual approaches when results are compared between clinicians.^7,8^ Automated segmentation processes have received increased attention with the capability of analyzing large datasets systematically without fatigue.^9^ Automated segmentation of MRI data typically falls into one of two categories: either atlas-based or artificial intelligence (AI)-based methods.^10^ Atlas-based techniques utilize either a hand-labeled or statistical atlas, which is registered to the scan being analyzed to identify neuroanatomical structures.^11^ AI-based segmentation processes are trained to analyze neuroanatomical structures based on labeled training data and are then deployed to analyze MRI scans.^12^ AI techniques are more flexible based on their training data and once trained may reduce the computational cost.^13^ However, AI approaches have not had widespread adoption at the time of this study, and currently approaches are more specialized.^14–18^

Similarly, atlas-based segmentation processes have their own challenges. Firstly, the atlases utilized in the most common MRI segmentation pipelines may not be representative of the population being analyzed. For instance, Freesurfer utilizes the Desikan-Killiany dataset with 40 neurotypical control participants between the ages of 19 and 86 years, which may limit interpretation of subjects who are outside of this age range or who might be impaired.^19^ Similarly, with the pediatric brain specifically, challenges arise including increased noise, reduced contrast between tissues, and ongoing myelination.^20^ Furthermore, some of the most utilized automated segmentation methods ignore smaller substructures like the hypothalamus,^21^ pons,^22^ pituitary gland,^23^ and optic nerve.^24^ Ultimately, it is important to investigate which atlas-based technique is suitable for each analysis. In this study, we aim to investigate some of the most frequently utilized atlas-based techniques including Freesurfer, FSL, volBrain, and SPM in GM1 gangliosidosis brains, to understand which pipeline provides the highest accuracy of volumetric measurements compared to a manual approach.

GM1 gangliosidosis is an inherited ultra-rare neurodegenerative lysosomal storage disorder caused by variants in the *GLB1* gene encoding β-galactosidase.^25^ Three clinical subtypes for GM1 exist based on the age of symptom onset and residual enzyme activity.^26^ Type I (infantile) GM1 is the most severe form of the disease with symptoms beginning in the first year of life and death often before the age of 3.^27^ Type II GM1 gangliosidosis can be further classified into two forms (late-infantile and juvenile) with the late-infantile form leading to symptom onset between age 1 and 2 and survival into the second decade. Juvenile GM1 patients have symptom onset between 3 and 5 years old with survival into the fourth decade.^28^ Type III (adult) GM1 gangliosidosis patients typically have manifesting symptoms in the second decade, slower disease progression, and the longest survival. Currently, there are no approved therapies for GM1, and the disease is uniformly fatal. However, investigations into adeno-associated virus (AAV) mediated gene therapy have shown promise in animal models and one clinical trial in Type I and II GM1 gangliosidosis patients is currently underway (ClinicalTrials.gov Identifier NCT03952637).^29,30^

**Figure 1.**
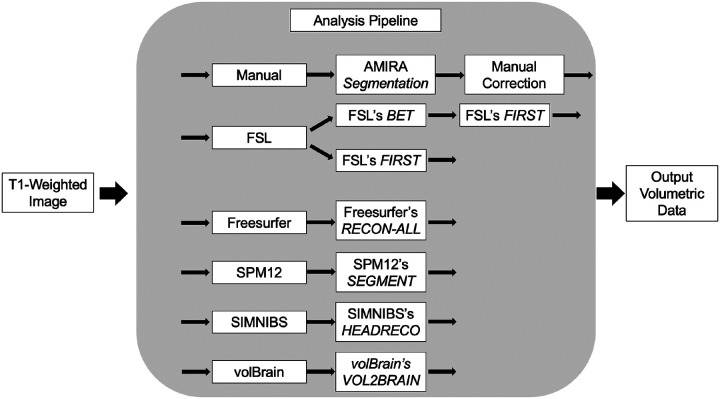
Segmentation Analysis Pipeline Overview. T1-Weighted MRI scans were processed through 5 fully automated segmentation pipelines in addition to the manual segmentation process. FSL required two different sub-pipelines. FSL’s *FIRST* was used to analyze bilateral volumes of the thalamus, caudate nucleus, and lentiform nucleus and FSL’s *FAST* was used to analyze whole brain volumes.

Previous investigations utilizing brain MRI in Type II GM1 gangliosidosis patients have demonstrated numerous findings.^31,32^ Atrophy of the cerebral cortex, corpus callosum, caudate nucleus, cerebellum, cerebellar white matter, basal ganglia, and associated ventricular enlargement have all been described.^31,32^ In this study, we first investigated the performance of 5 different fully automated MRI segmentation pipelines compared to a manual process in identifying MRI pathogenesis in GM1 gangliosidosis patients (Figure 1). Second, we evaluated which automated pipeline gave the most accurate results in terms of volumetric analysis of the different brain structures.

## Methods

### Type 2 GM1 Gangliosidosis Participants

**Figure 2.**
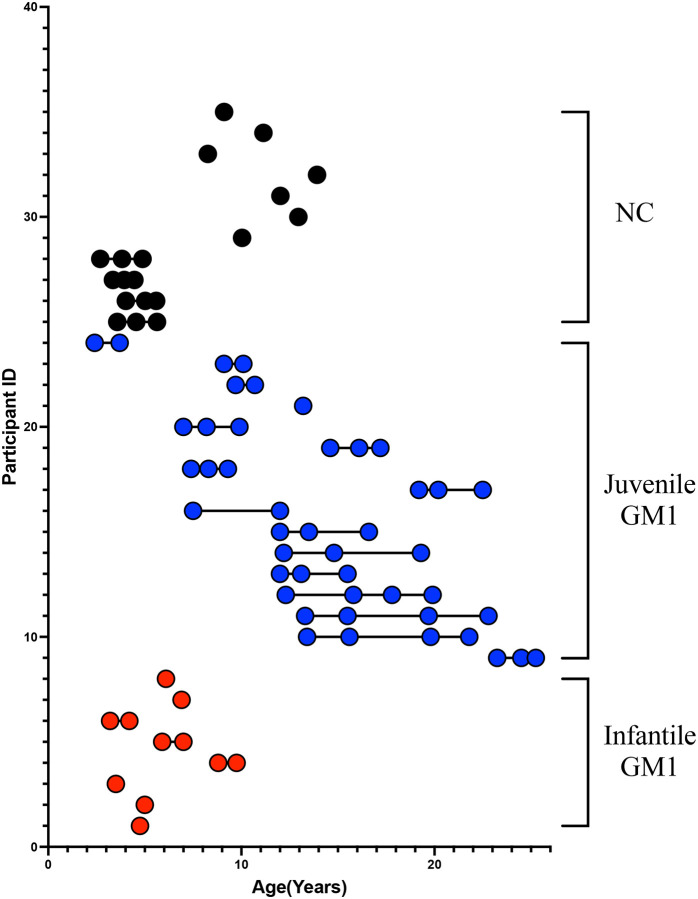
Age stratification of each participant at T1-weighted MRI scan. Infantile GM1 patients are shown in Red. Juvenile GM1 patients are shown in blue. Neurotypical controls are shown in black. Connecting lines indicate participant had repeated MRI scans.

Twenty-four GM1 patients from the “Natural History of Glycosphingolipid Storage Disorders and Glycoprotein Disorders” (ClinicalTrials.gov ID: NCT00029965) were included in this study.^33^ As described in D’Souza et al, a GM1 diagnosis was made through β-galactosidase enzyme deficiency or by biallelic variants in *GLB1*.^25^ Forty-five MRI scans from 16 juvenile (baseline age: 11.8 ± 4.9 years) patients were included in this study alongside 11 MRI scans from 8 late-infantile patients (Figure 2, baseline age: 5.5 ± 1.8 years).

### Neurotypical Controls

Neurotypical early childhood control MRI scans were gathered from Open Science Framework and included participants from the “Calgary Preschool magnetic resonance imaging (MRI) dataset” and consisted of participants between 2 and 8 years of age from the University of Calgary.^34^ Late childhood and adolescent neurotypical control MRI were gathered from Figshare and included participants from the “Detailing neuroanatomical development in late childhood and early adolescence using NODDI” study and consisted of participants between 8 and 13 years of age also from the University of Calgary.^35^ In total 19 MRI scans were obtained, from 11 neurotypical controls (Figure 2, baseline age: 8.3 ± 4.2 years).

### T1-Weighted MRI Acquisition

MRI scans were performed on a Philips 3T system (Achieva, Philips Healthcare, Best, The Netherlands) for all GM1 patients under sedation at all time-points with an 8-channel SENSE head coil. Images were acquired using a 3D T1-weighted protocol with slice thickness of 1 mm, repetition time (TR) of 11 ms, and an echo time (TE) of 7 ms. Unprocessed digital imaging and communications in medicine (DICOM) images were converted to NIfTI using *dcm2niix*.^36^ Calgary preschool and adolescent neurotypical controls were scanned without sedation on a General Electric 3T system (MR750w, GE Healthcare, Chicago, IL, USA) using a 32-channel head coil.^34,35^ T1-weighted imaging from preschool neurotypical controls was acquired with TR/TE = 8.23/3.76 ms and resolution of 0.9 mm × 0.9 mm × 0.9 mm (resampled to 0.45 mm × 0.45 mm × 0.9 mm).^34^ T1-weighted imaging from adolescent Calgary neurotypical controls was acquired with TR/TE = 8.21/3.16 ms and 0.8 mm^3^ isotropic resolution.^35^

### Manual MRI Volumetric Segmentation

Volumetric segmentations were performed on the 3D T1-weighted images, which provided sufficient tissue contrast to visualize the structures of interest. A semi-automated approach was used with manual corrections for the larger structures and a manual process was implemented for the smaller structures as previously described Zoppo et al.^8^ The following structures were segmented on all the scans: whole brain (without ventricles), cerebellum, ventricles, corpus callosum, caudate, lentiform nucleus, and thalamus. In brief, a team of researchers and trained neuroradiologists used the AMIRA analysis software (Amira, Thermo Fischer Scientific, Waltham, MA) in the native image space to define the regions of interest (ROIs) around the boundaries of the structures using a combination of signal thresholding and/or manual demarcation on a slice-by-slice basis. The method for determining the segmentation boundaries of each structure was carried out as described in prior work.^8^ The ROIs from all slices were rendered into a 3D volume for each structure to estimate the structure volume.

### Freesurfer Volumetric Segmentation

Automated segmentation of MRI images was first performed using Freesurfer’s (v7.4.1) *recon-all* reconstruction pipeline to calculate volumes for the structures of interest.^37–45^ Volumes of the whole brain (sum of gray matter and white matter), ventricles, cerebellum, caudate, thalamus, lentiform nucleus (sum of the globus pallidus and putamen), and corpus callosum were calculated, corresponding to the manual segmentation process. Automated segmentation was performed post-hoc of the manual segmentation process.

### FSL Volumetric Segmentation

MRI data was also segmented using tools from the FMRIB Software Library (FSL).^46–48^ T1-weighted imaging was first sent through FSL’s *bet* for brain extraction. Brain extracted images were then analyzed using FSL’s *fast* to create partial volume maps of the cerebrospinal fluid, gray matter, and white matter. T1-weighted imaging was also sent through FSL’s *run_first_all* pipeline to segment subcortical structures including the bilateral thalamus, bilateral putamen, bilateral globus pallidus, and bilateral caudate nucleus. FSL’s *fslstats* was then used to calculate volumes from the partial volume maps and segmented images.^46–48^

### volBrain Volumetric Segmentation

Automated segmentation of MRI images was also performed using volBrain’s updated segmentation algorithm, vol2Brain.^49^ vol2Brain uses the preprocessing algorithm from the original volBrain pipeline, followed by segmentation.^50^ T1-weighted images in this study were uploaded to the volBrain server, and volumetric results were obtained for the intracranial volume, bilateral gray matter, bilateral white matter, cerebellum, bilateral thalamus, bilateral globus pallidus, bilateral putamen, and bilateral caudate nucleus.

### SPM Volumetric Segmentation

Automated segmentation of T1-weighted images was also performed using Statistical Parametric Mapping (SPM) 12 housed within MATLAB R2023a (The MathWorks Inc., Natick, MA, USA).^51^ Tissue probability maps of the cerebrospinal fluid, gray matter, and white matter were created using the Segment tool. FSL’s *fslstats* was then used to calculate volumes from the tissue probability maps.

### Headreco Volumetric Segmentation

T1-weighted imaging in this study was also sent through SimNIBS’ (v3.2.6) *headreco* segmentation pipeline.^52^ *Headreco* utilizes SPM12 followed by the Computational Anatomy Toolbox (CAT12) to improve segmentation by SPM12.^51–54^ Gray matter, white matter, and cerebrospinal fluid volumes were calculated from the final mask generated by *headreco* using *fslstats*. Ventricle volume was also calculated using *fslstats* from the ventricle mask generated by *headreco*. Masks for the bilateral thalamus, corpus callosum, and cerebellum were generated from CAT12 after deformation-based morphometry (DBM), registration to the IXI-database^55^ of 555 healthy participants, and the Hammers atlas^56^ was used to select ROIs. Volumes of the bilateral thalamus, corpus callosum, and cerebellum were calculated from each respective mask using *fslstats*.

### Statistical Analysis

Cross-sectional data was analyzed in GraphPad Prism (v10.1.0, GraphPad Software, Boston, MA, USA) using Welch’s 2 sample t-test to demonstrate differences between neurotypical controls and both juvenile and late-infantile GM1 patients. Neurotypical controls were selected to age-match the late-infantile (Supplement B) and juvenile (Supplement C) GM1 patients separately since the juvenile patients were older than the late-infantile patients. Linear regression modeling was performed in GraphPad Prism to calculate correlation coefficients (R^2^) and F-statistics; *p*-values from the F-statistics were compared between each automated segmentation process and the manual segmentation process for the 7 structures of interest.

## Results

### Whole Brain Volume

**Figure 3.**
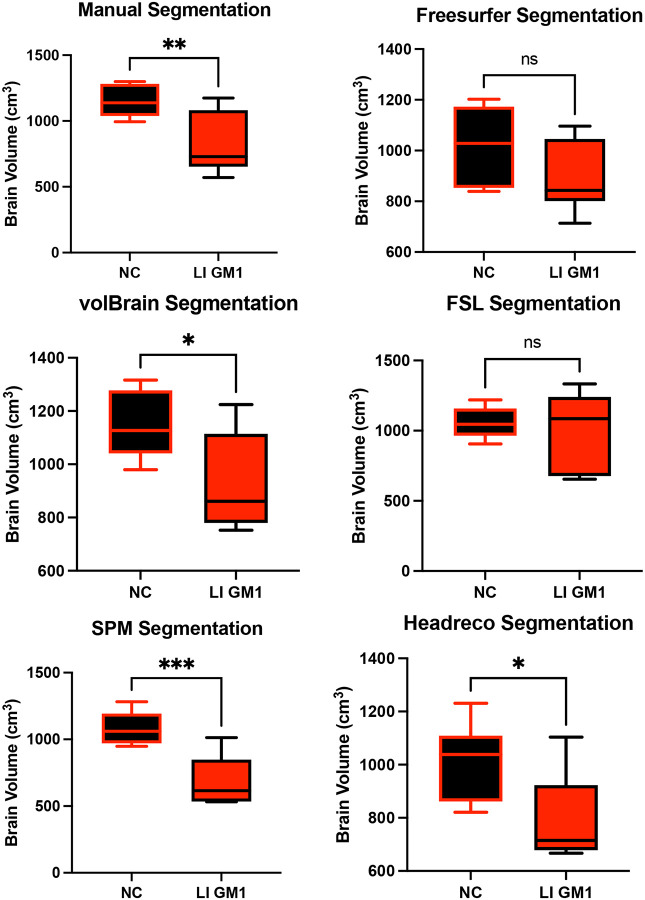
Cross-sectional evaluation of the 5 automated segmentation algorithms to demonstrate cohort differences in total brain volume. Late-infantile (LI) GM1 patients are shown in red. Neurotypical controls (NC) are shown in black. *P*-values were calculated from the *t*-statistic. * *P* < 0.05, ** *P* < 0.01, *** *P* < 0.001, **** *P* < 0.0001.

**Figure 4.**
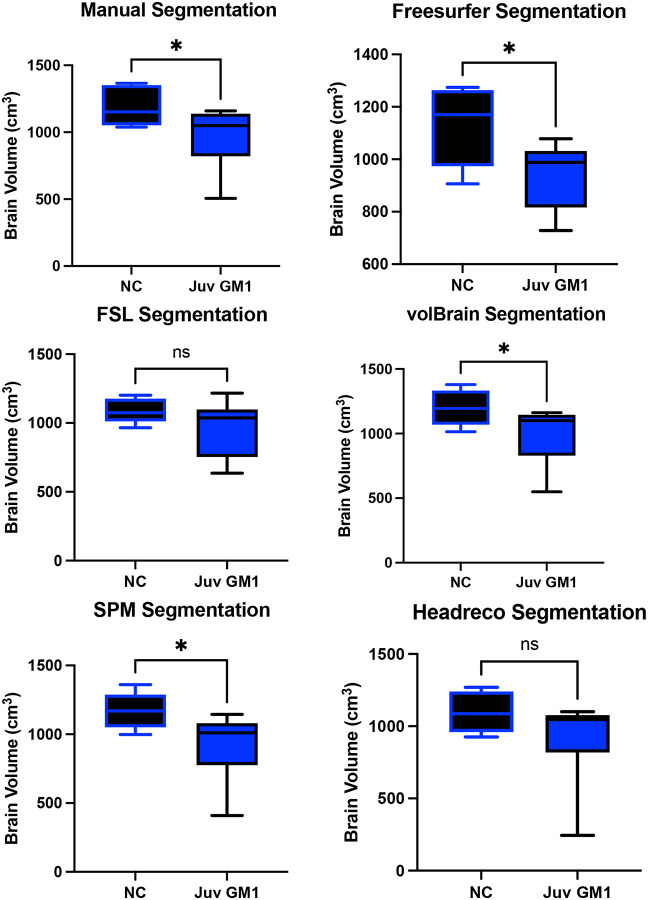
Cross-sectional evaluation of 5 automated segmentation algorithms ability to demonstrate cohort differences in total brain volume. Juvenile (Juv) GM1 patients are shown in blue. Neurotypical controls (NC) are shown in black. *P*-values were calculated from the *t*-statistic. * *P* < 0.05, ** *P* < 0.01, *** *P* < 0.001, **** *P* < 0.0001.

Manual whole brain volume segmentation demonstrated higher whole brain volume in neurotypical controls when compared to both late-infantile (*t*(12) = 3.47, *p* = 0.0047, Figure 3) and juvenile (*t*(19) = 2.87, *p* = 0.0101, Figure 4) GM1 patients. volBrain, SPM, and Headreco also demonstrated higher whole brain volume in neurotypical controls compared to late-infantile patients; however, Freesurfer and FSL did not find a significant difference between late-infantile patients and neurotypical controls. Freesurfer, volBrain, and SPM demonstrated higher whole brain volume in neurotypical controls compared to juvenile patients; however, FSL and Headreco did not find a significant difference between the two cohorts (Figure 4).

**Figure 5.**
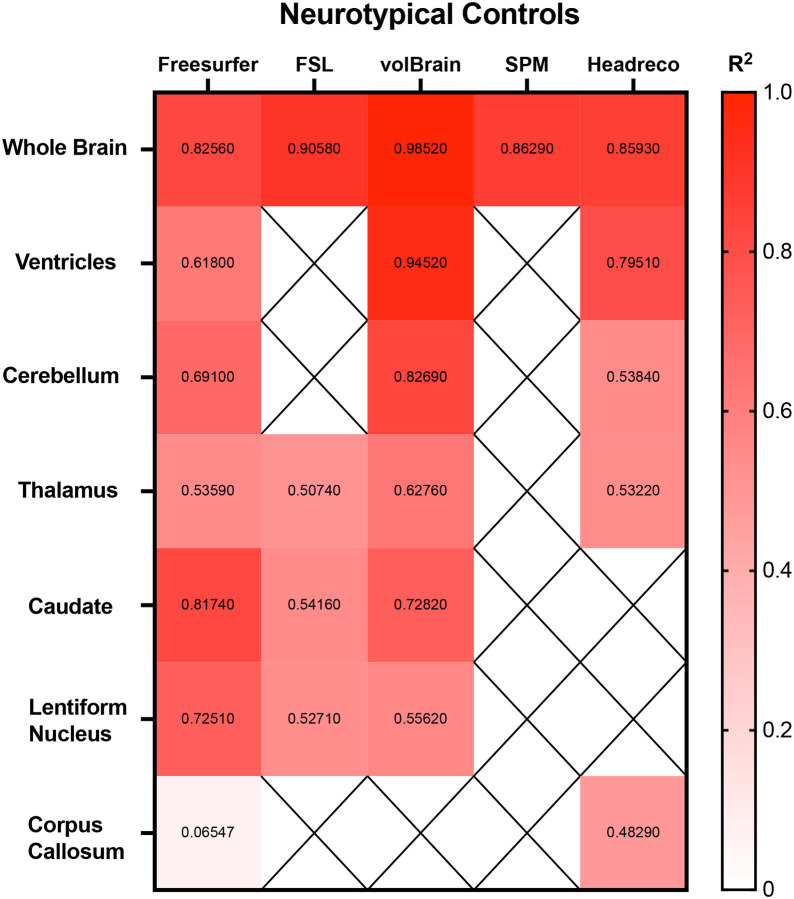
Heatmap of correlations strengths (R^2^) between the manual segmentation process and the 5 fully automated pipelines for the 7 structures of interest in neurotypical controls. N/A are designations where the region was not calculated using the specified segmentation algorithm.

Correlations between the 5 fully automated segmentation processes and our manual process showed volBrain (R^2^ = 0.9852) and FSL (R^2^ = 0.9058) with R^2^ values above 0.90 in neurotypical controls (Figure 5). In juvenile GM1 patients, volBrain (R^2^ = 0.9583), Freesurfer (R^2^ = 0.9196), and SPM (R^2^ = 0.9642) all had R^2^ values above 0.90 (Figure 8). In late-infantile GM1 patients, volBrain (R^2^ = 0.9169) was the only fully automated segmentation process with an R^2^ above 0.90 when compared to the manual process (Figure 9).

### Ventricle Volume

Manual ventricle volumetric segmentation demonstrated higher ventricle volume in late-infantile GM1 patients when compared to neurotypical controls (*t*(12) = 2.98, *p* = 0.0115, Figure 8). Freesurfer, volBrain, and Headreco were also able to demonstrate this difference finding enlargement of the ventricles in late-infantile GM1 patients (Figure 8). In juvenile GM1 patients, no statistical difference was found in ventricle volume when compared to neurotypical controls using manual segmentation (*t*(19) = 2.98, *p* = 0.0115, Supplement Figure F1). Freesurfer, volBrain, and Headreco also did not find a statistical difference in ventricle volume between juvenile GM1 patients and neurotypical controls (Supplement Figure F1).

**Figure 6.**
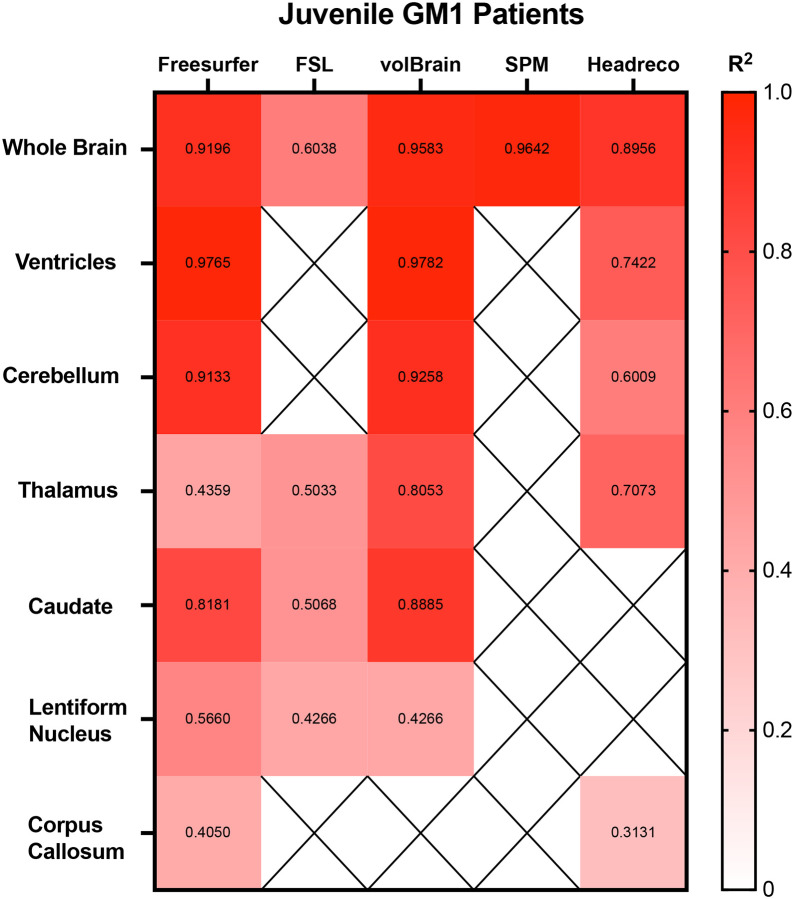
Heatmap of correlations strengths (R^2^) between the manual segmentation process and the 5 fully automated pipelines for the 7 structures of interest in juvenile (Juv) GM1 patients. N/A are designations where the region was not calculated using the specified segmentation algorithm.

**Figure 7.**
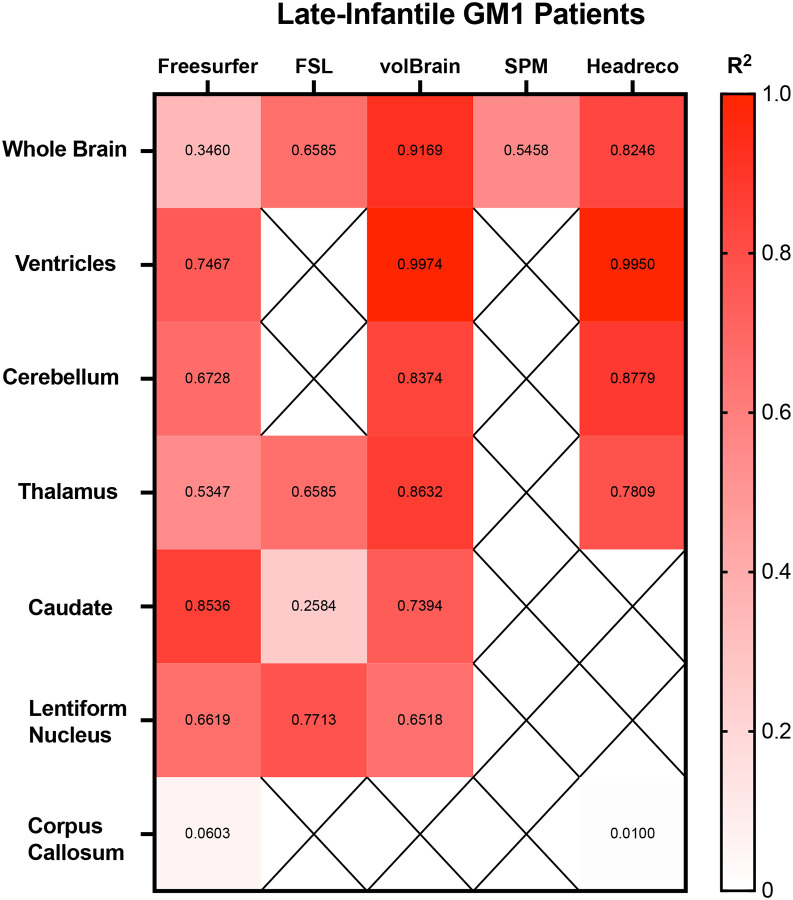
Heatmap of correlations strengths (R^2^) between the manual segmentation process and the 5 fully automated pipelines for the 7 structures of interest in late-infantile (LI) GM1 patients. N/A are designations where the region was not calculated using the specified segmentation algorithm.

**Figure 8.**
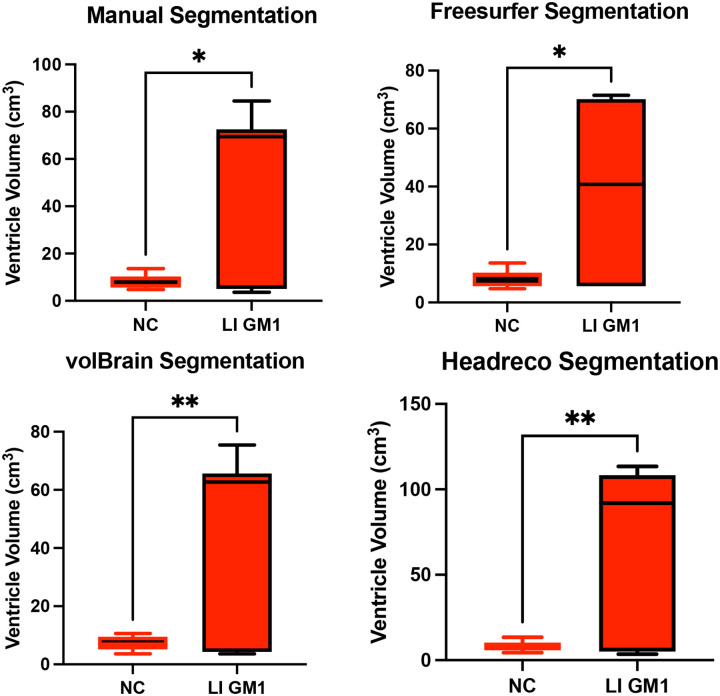
Cross-sectional evaluation of 3 automated segmentation algorithms to demonstrate cohort differences in ventricle volume. Late-infantile (LI) GM1 patients are shown in red. Neurotypical controls (NC) are shown in black. *P*-values were calculated from the *t*-statistic. * *P* < 0.05, ** *P* < 0.01, *** *P* < 0.001, **** *P* < 0.0001.

Correlations between the three fully automated ventricle segmentation processes and our manual process showed volBrain (R^2^ = 0.9452) as the only pipeline with a R^2^ value above 0.90 in neurotypical controls (Figure 5). In juvenile GM1 patients, volBrain (R^2^ = 0.9782) and Freesurfer (R^2^ = 0.9765) both had R^2^ values above 0.90 (Figure 6). In late-infantile GM1 patients, volBrain (R^2^ = 0.9974) and Headreco (R^2^ = 0.9950) both had R^2^ above 0.90 when compared to the manual process (Figure 7).

### Cerebellar Volume

Manual cerebellar volume segmentation demonstrated higher cerebellar volume in neurotypical controls when compared to both late-infantile (t(12) = 3.32, *p* = 0.0061, Supplement Figure E1) and juvenile (*t*(19) = 2.69, *p* = 0.0146, Supplement Figure F2) GM1 patients. In late-infantile GM1 patients, Freesurfer, volBrain, and Headreco also demonstrated this result, finding larger cerebellar volume in neurotypical controls (Supplement Figure E1). In juvenile GM1 patients, Freesurfer and volBrain also demonstrated this result finding larger cerebellar volume in neurotypical controls, however Headreco did not find a statistical difference between juvenile GM1 patients and neurotypical controls (Supplement Figure F2).

Correlations between the three fully automated cerebellar segmentation processes and our manual process showed volBrain (R^2^ = 0.8259) as the only pipeline with a R^2^ value above 0.80 in neurotypical controls (Figure 5). In juvenile GM1 patients, volBrain (R^2^ = 0.9258) and Freesurfer (R^2^ = 0.9133) both had R^2^ values above 0.90 (Figure 6). In late-infantile GM1 patients, volBrain (R^2^ = 0.8374) and Headreco (R^2^ = 0.8779) both had R^2^ above 0.80 when compared to the manual process (Figure 7).

### Thalamic Volume

**Figure 9.**
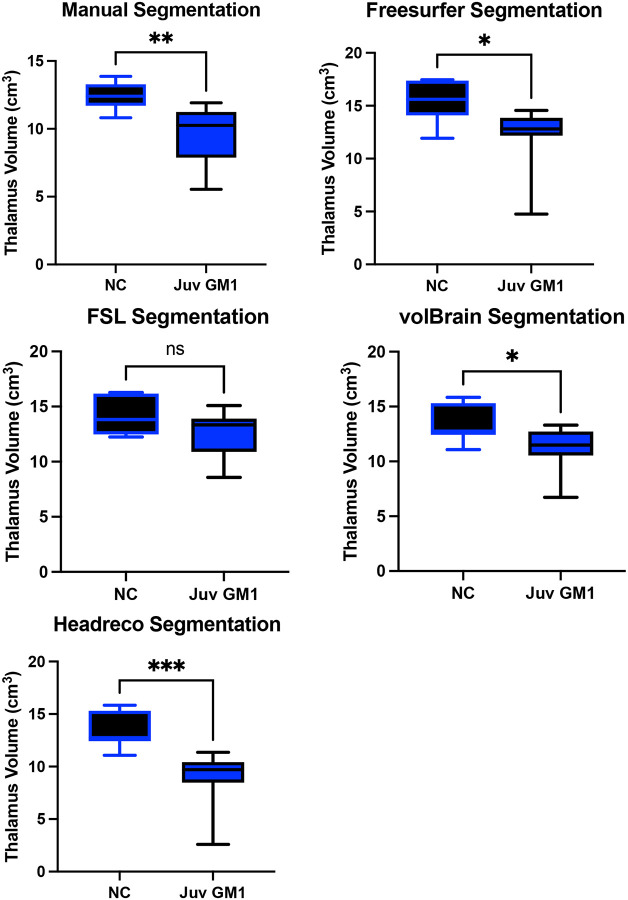
Cross-sectional evaluation of 4 automated segmentation algorithms ability to demonstrate cohort differences in thalamic volume. Juvenile (Juv) GM1 patients are shown in blue. Neurotypical controls (NC) are shown in black. *P*-values were calculated from the *t*-statistic. * *P* < 0.05, ** *P* < 0.01, *** *P* < 0.001, **** *P* < 0.0001.

Manual thalamic volume segmentation demonstrated higher thalamic volume in neurotypical controls when compared to both late-infantile (t(12) = 6.14, *p* < 0.0001, Supplement Figure E2) and juvenile (t(19) = 3.51, *p* = 0.0023, Figure 9) GM1 patients. In late-infantile GM1 patients, Freesurfer, FSL, volBrain, and Headreco also demonstrated this result, finding larger thalamic volume in neurotypical controls (Supplement Figure E2). In juvenile GM1 patients, Freesurfer, volBrain, and Headreco also demonstrated this result finding larger thalamic volume in neurotypical controls, however FSL did not find a statistical difference between juvenile GM1 patients and neurotypical controls (Figure 9).

Correlations between the four fully automated thalamic segmentation processes and our manual process showed volBrain (R^2^ = 0.6276) with the highest R^2^ value neurotypical controls (Figure 5). In juvenile GM1 patients, volBrain (R^2^ = 0.8053) again had the highest R^2^ value (Figure 6). In late-infantile GM1 patients, volBrain (R^2^ = 0.8632) again had the highest R^2^ value when compared to the manual process (Figure 7).

### Caudate Volume

Manual caudate volume segmentation demonstrated higher thalamic volume in neurotypical controls when compared to both late-infantile (t(12) = 2.72, *p* = 0.0185, Supplement Figure E3) and juvenile (t(19) = 6.00, *p* < 0.0001, Supplement Figure F3) GM1 patients. In late-infantile GM1 patients, Freesurfer, FSL, and volBrain also demonstrated this result, finding larger caudate volume in neurotypical controls (Supplement Figure E3). In juvenile GM1 patients, Freesurfer also demonstrated this result finding larger caudate volume in neurotypical controls, however FSL and volBrain did not find a statistical difference between juvenile GM1 patients and neurotypical controls (Supplement Figure F3).

Correlations between the three fully automated caudate nucleus segmentation processes and our manual process showed Freesurfer (R^2^ = 0.8174) with the highest R^2^ value in neurotypical controls (Figure 5). In juvenile GM1 patients, volBrain (R^2^ = 0.8885) had the highest R^2^ value (Figure 6). In late-infantile GM1 patients, Freesurfer (R^2^ = 0.8536) again had the highest R^2^ value when compared to the manual process (Figure 7).

### Lentiform Nucleus Volume

Manual caudate volume segmentation demonstrated higher thalamic volume in neurotypical controls when compared to both late-infantile (t(12) = 4.46, *p* = 0.0008, Supplement Figure E4) and juvenile (t(19) = 7.51, *p* < 0.0001, Supplement Figure F4) GM1 patients. In late-infantile GM1 patients, Freesurfer, FSL, and volBrain also demonstrated this result, finding larger lentiform nucleus volume in neurotypical controls (Supplement Figure E4). In juvenile GM1 patients, Freesurfer, FSL, and volBrain also demonstrated this result finding larger lentiform nucleus volume in neurotypical controls (Supplement Figure F4).

Correlations between the three fully automated lentiform nucleus segmentation processes and our manual process showed Freesurfer (R^2^ = 0.7251) with the highest R^2^ value in neurotypical controls (Figure 5). In juvenile GM1 patients, volBrain (R^2^ = 0.6605) had the highest R^2^ value (Figure 6). In late-infantile GM1 patients (Figure 7), Freesurfer (R^2^ = 0.6619) again had the highest R^2^ value when compared to the manual process. In late-infantile GM1 patients, volBrain had a similar correlation coefficient strength to Freesurfer (0.6518).

### Corpus Callosum Volume

Manual corpus callosum volume segmentation demonstrated higher corpus callosum volume in neurotypical controls when compared to both late-infantile (t(12) = 4.46, *p* = 0.0008, Supplement Figure E5) and juvenile (t(19) = 2.49, *p* = 0.0222, Supplement Figure F5) GM1 patients. In late-infantile GM1 patients, Freesurfer also demonstrated this result, finding larger corpus callosum volume in neurotypical controls (Supplement Figure E5), however Headreco did not demonstrate this difference. In juvenile GM1 patients, Headreco also demonstrated this result finding larger corpus callosum volume in neurotypical controls (Supplement Figure F5), however Freesurfer did not demonstrate this difference.

Correlations between the two fully automated corpus callosum segmentation processes and our manual process showed SimNIBS’ Headreco (R^2^ = 0.4829) with the highest R^2^ value neurotypical controls (Figure 5). In juvenile GM1 patients, Freesurfer (R^2^ = 0.4050) again had the highest R^2^ value (Figure 6). In late-infantile GM1 patients, both Freesurfer and Headreco had weak correlations (R^2^ < 0.1) with the manual process (Figure 7).

### Extended Results

Individual figures for the comparisons between late-infantile and juvenile with neurotypical controls for the remaining analyzed structures are included in sections E and F of the Supplementary materials, respectively. Individual figures for the correlations between each of the 5 automated pipelines with the manual process for all analyzed structures are included in sections H–L of the Supplementary Materials. Slope estimates and y-intercept values with standard errors for the correlations between each of the 5 automated pipelines with the manual process are included in section M of the Supplementary Materials.

## Discussion

This study assessed the capabilities of 5 fully automated brain MRI segmentation pipelines compared to a manual approach in juvenile and infantile GM1 gangliosidosis patients and neurotypical controls. We aimed to demonstrate the accuracy of the fully automated techniques, which have significant time saving advantages and can enable the analysis of large-scale datasets and larger complex brain structures including the cerebral white matter. We found our automated results to closely reflect those found by the manual approach suggesting the utility of fully automated processes in neurodegenerative diseases.

We first compared each automated segmentation pipeline’s ability to differentiate between GM1 patients and neurotypical controls using the 7 brain structures of interest. The manual segmentation process found statistically significant differences in 6 out of the 7 brain structures between juvenile GM1 patients and neurotypical controls. For the specific segmentations that were performed by each of the fully automated pipelines, statistically significant differences in brain structure volume were observed in 5 out of 6 for Freesurfer, 4 out of 5 for volBrain, 1 out of 4 for FSL, 1 out of 1 for SPM12, and 2 out of 4 for Headreco. In late-infantile patients, the manual segmentation process found statistically significant differences in all 7 brain structures. Freesurfer, volBrain, FSL, SPM12, and Headreco identified statistically significant differences in 6 out of 7, 6 out of 6, 3 out of 4, 1 out of 1, and 4 out of 5 brain structure volumes, respectively.

Correlation measurements were then performed comparing the volumetric results from each of the fully automated pipelines with the results from the manual approach to understand how they relate to a clinician’s measurements. volBrain showed the strongest correlation with the manual pipeline for the whole brain, ventricles, and thalamus for all three cohorts (Figures 5, 6, and 7). volBrain also showed the strongest correlation in the cerebellum in both the neurotypical controls and juvenile GM1 patients, with Headreco demonstrating the strongest correlation in the late-infantile GM1 patients. Freesurfer showed the strongest correlation with the manual pipeline for the caudate nucleus in neurotypical controls and late-infantile GM1 patients, with volBrain demonstrating the strongest correlation in the juvenile GM1 patients. Freesurfer also showed the strongest correlation for the lentiform nucleus in the neurotypical controls, with FSL demonstrating the strongest correlation in the late-infantile GM1 patients, and volBrain demonstrating the strongest correlation in the juvenile GM1 patients. Overall, our results suggest that among the 5 automated pipelines, volBrain is the most accurate for analyzing ventricle size, total brain volume, and thalamic volume in GM1 patients while Freesurfer is the most accurate for analyzing the caudate nucleus.

Corpus callosum segmentation in this study resulted in varying success. On the one hand, Freesurfer was able to demonstrate the correct positive relationship in the late-infantile GM1 patients and Headreco in juvenile GM1 patients. However, correlations with the manual process were weak and highly variable for all three cohorts. Furthermore, variability in the measured size was also observed. Headreco appears to measure further into the bilateral hemispheres while Freesurfer appears to exclude relevant regions that is included in our manual process. This result is consistent with previous analysis which suggests that a dedicated corpus callosum segmentation process may be justified for future studies.^57–59^

Limitations of this study need to be considered before the results are used to guide clinical practice. First, this study is limited by a small sample size in neurotypical controls (n = 11, 19 MRI scans), juvenile GM1 patients (n = 16, 45 MRI scans), and late-infantile patients (n = 8, 11 MRI scans). However, this study represents the largest MRI dataset of Type II GM1 gangliosidosis patients.^60^ Second, this study is limited by the variations in scanners and protocols used between the GM1 patients and neurotypical controls. However, all 6 analysis techniques were given the same images to analyze, and the results of this study focus on the comparisons between these techniques. Ultimately, future investigations should utilize identical protocols when comparing MRI segmentation techniques. Dice similarity coefficients, Hausdorff distance, and the mean distance to agreement were also not considered in this study.^61–63^ Future studies investigating the accuracy of automated MRI segmentation methods should incorporate these metrics, however in this pilot study we focused on the capability of these methods for demonstrating GM1 associated neurodegeneration compared to neurotypical controls.

## Conclusion

In this study, we compared 5 fully automated segmentation pipelines with a manual pipeline in neurotypical controls and juvenile and late-infantile GM1 gangliosidosis patients. We analyzed 7 brain structures of interest including volumes of the total brain, cerebellum, ventricles, bilateral thalami, bilateral lentiform nucleus, bilateral caudate nucleus, and corpus callosum. We found that a combination of pipelines led to the strongest correlations with the manual pipeline with volBrain’s *vol2Brain* demonstrating the strongest correlations for the whole brain, ventricles, and thalamus, Freesurfer’s *recon-all* demonstrating the strongest correlations for the caudate nucleus, and a combination of pipelines demonstrating the strongest correlations for the cerebellum and lentiform nucleus. Ultimately, we found our fully automated results to be consistent with our manual process, however further studies are needed to investigate other neurodegenerative and lysosomal storage disorders.

## Supplementary Material

1

## Data Availability

The data described in this manuscript are available from the corresponding author upon reasonable request. Neuroimaging data for the early childhood neurotypical control group are publicly available here: https://osf.io/axz5r/.^33^ Neuroimaging data for the adolescent neurotypical control group are publicly available here: https://doi.org/10.6084/m9.figshare.6002273.v2.^34^
