# Supplementary material for: A Case for Automated Segmentation of MRI Data in Milder Neurodegenerative Diseases
Source: medRxiv. 2025 Feb 20:2025.02.18.25322304. Preprint. [Version 1] doi: 10.1101/2025.02.18.25322304 (PMC11875249; doi:10.1101/2025.02.18.25322304)
Supplement: 1 [file NIHPP2025.02.18.25322304V1-supplement-1.pdf]

medRxiv preprint doi: https://doi.org/10.1101/2025.02.18.25322304; this version posted February 20, 2025. The copyright holder for this preprint (which was not certified by peer review) is the author/funder, who has granted medRxiv a license to display the preprint in perpetuity.
This article is a US Government work. It is not subject to copyright under 17 USC 105 and is also made available for use under a CC0 license.

A Case for Automated Segmentation of MRI in Milder Neurodegenerative Diseases: Supplementary Material

Table of Contents

Methods……………………………………………………………………………………27

Supplement A: Natural History Study Participant Characteristics…..…………..……...………..27

Supplement B: Late-Infantile GM1 Patients Age Matched Methodology………………………..28

Supplement C: Juvenile GM1 Patients Age Matched Methodology……………………………...29

Supplement D: MRI Scan Exclusion……………………………………………………………..31

Results…………………………………………………………………………………….......34

Supplement E. Cross-sectional analysis of Late-Infantile Patients………………………………34

Supplement F. Cross-sectional analysis of Juvenile Patients……………………………………..…39

Supplement G. Correlation Strength Tables between Manual and Automated Segmentation……44

Supplement H: Correlations between Manual and Freesurfer Segmentation……………….………47

Supplement I: Correlations between Manual and volBrain Segmentation……………...…….…..50

Supplement J: Correlations between Manual and FSL Segmentation………………...……...…..53

Supplement K: Correlations between Manual and SPM Segmentation…………….……………56

Supplement L: Correlations between Manual and Headreco Segmentation……………..………58

Supplement M: Correlation Data Slope Estimates and Intercepts…………………….…………61

Supplementary References……...………………………………………………………...63

medRxiv preprint doi: https://doi.org/10.1101/2025.02.18.25322304; this version posted February 20, 2025. The copyright holder for this preprint
(which was not certified by peer review) is the author/funder, who has granted medRxiv a license to display the preprint in perpetuity.
This article is a US Government work. It is not subject to copyright under 17 USC 105 and is also made available
for use under a CC0 license.

Supplementary Methods

Supplement A: Natural History Study Participant Characteristics[1]

Table A1. Natural History Study T1-Weighted MRI Cohort (n = 24), specific ages redacted per MedArXiv requirements

Participant	GM1 Sub-type	Baseline Age (years)	Scan #2 Age (years)	Scan #3 Age (years)	Scan #4 Age (years)	Average interval between imaging (years)	Number of T1 Scans
NHS 13	LI	0-5	N/A	N/A	N/A	N/A	1
NHS 24	LI	6-10	N/A	N/A	N/A	N/A	1
NHS 35	LI	0-5	N/A	N/A	N/A	N/A	1
NHS 63	LI	0-5	N/A	N/A	N/A	N/A	1
NHS 72	LI	6-10	6-10	N/A	N/A	0.95	2
NHS 73	LI	0-5	6-10	N/A	N/A	1.1	2
NHS 84	LI	0-5	0-5	N/A	N/A	1.0	2
NHS 85	LI	6-10	N/A	N/A	N/A	N/A	1
NHS 03	Juv	21-25	21-25	21-25	N/A	1.0	3
NHS 09	Juv	11-15	11-15	16-20	21-25	2.8	4
NHS 10	Juv	11-15	11-15	16-20	21-25	3.2	4
NHS 11	Juv	11-15	16-20	16-20	16-20	2.5	4
NHS 20	Juv	11-15	11-15	11-15	N/A	1.8	3
NHS 25	Juv	11-15	11-15	16-20	N/A	3.6	3
NHS 26	Juv	11-15	11-15	16-20	N/A	2.3	3
NHS 27	Juv	6-10	11-15	N/A	N/A	4.5	2
NHS 28	Juv	16-20	16-20	21-25	N/A	1.7	3
NHS 54	Juv	6-10	6-10	6-10	N/A	1.0	3
NHS 58	Juv	11-15	16-20	16-20	N/A	1.3	3
NHS 69	Juv	6-10	6-10	6-10	N/A	1.5	3
NHS 71	Juv	11-15	N/A	N/A	N/A	N/A	1
NHS 93	Juv	5-10	5-10	N/A	N/A	1.0	2
NHS 95	Juv	5-10	5-10	N/A	N/A	1.0	2
NHS 95	Juv	0-5	0-5	N/A	N/A	1.3	2

medRxiv preprint doi: https://doi.org/10.1101/2025.02.18.25322304; this version posted February 20, 2025. The copyright holder for this preprint (which was not certified by peer review) is the author/funder, who has granted medRxiv a license to display the preprint in perpetuity. This article is a US Government work. It is not subject to copyright under 17 USC 105 and is also made available for use under a CC0 license.

Supplement B: Age-Matched Methodology for Late-Infantile Patients

To evaluate the 5 automated segmentation pipelines' ability to demonstrate volumetric differences between the late-infantile patients and neurotypical controls, we performed a cross-sectional analysis including age and sex-matched data (Table B1 and B2). Seven late-infantile patients' (4 females, 3 males) baseline MRI scans and 7 neurotypical control (4 females, 3 males) scans from the Calgary Preschool[2] and Adolescent[3] databases were included in this analysis.

Table B1. Late-infantile Age Matched Cohort

Participant	Sex	Age
NHS 13	F	0-5
NHS 35	F	0-5
NHS 63	M	0-5
NHS 72	M	6-10
NHS 73	M	0-5
NHS 84	F	0-5
NHS 85	F	6-10
		5.3 ± 1.9

Table B2. Late-infantile Age Matched Cohort of Neurotypical Controls

Participant	Dataset	Sex	Age
Calgary 10014	Preschool	M	0-5
Calgary 10027	Preschool	F	0-5
Calgary 10044	Preschool	M	0-5
Calgary 10064	Preschool	F	6-10
Calgary 15003	Adolescent	F	6-10
Calgary 15008	Adolescent	F	11-15
Calgary 15010	Adolescent	M	6-10
			6.0 ± 3.4

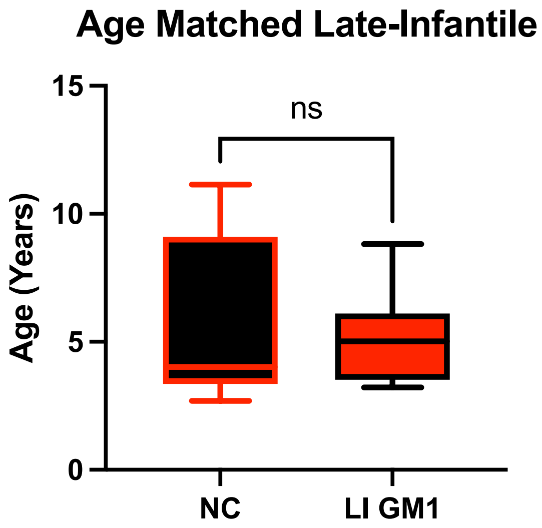

Figure B1. Age and sex-matched late-infantile GM1 gangliosidosis cohort evaluated for differences by a 2 tailed t-test (t=0.4771, df =12, p = 0.6418).

medRxiv preprint doi: https://doi.org/10.1101/2025.02.18.25322304; this version posted February 20, 2025. The copyright holder for this preprint (which was not certified by peer review) is the author/funder, who has granted medRxiv a license to display the preprint in perpetuity. This article is a US Government work. It is not subject to copyright under 17 USC 105 and is also made available for use under a CC0 license.

Supplement C: Age-Matched Methodology for Juvenile Patients

To evaluate the 5 automated segmentation pipelines' ability to demonstrate volumetric differences between the juvenile patients and neurotypical controls, we performed a cross-sectional analysis including age and sex-matched data (Table C1 and C2). Fourteen juvenile patients' (8 females, 6 males) baseline MRI scans and 7 neurotypical control (4 females, 3 males) scans from the Calgary Preschool[2] and Adolescent[3] databases were included in this analysis.

Table C1. Juvenile Age Matched Cohort

Participant	Sex	Age
NHS 09	M	11-15
NHS 10	F	11-15
NHS 11	M	11-15
NHS 20	F	11-15
NHS 25	F	11-15
NHS 26	M	11-15
NHS 27	M	6-10
NHS 28	F	16-20
NHS 54	F	6-10
NHS 58	F	11-15
NHS 69	F	6-10
NHS 93	F	6-10
NHS 94	M	6-10
NHS 95	M	0-5
		10.9 ± 4.1

Table C2. Juvenile Age Matched Cohort of Neurotypical Controls

Participant	Dataset	Sex	Age
Calgary 10027	Preschool	F	0-5
Calgary 14006	Preschool	M	6-10
Calgary 14008	Preschool	M	11-15
Calgary 14011	Preschool	M	11-15
Calgary 14013	Adolescent	F	11-15
Calgary 15003	Adolescent	F	6-10
Calgary 15008	Adolescent	F	11-15
			10.6 ± 2.9

medRxiv preprint doi: https://doi.org/10.1101/2025.02.18.25322304; this version posted February 20, 2025. The copyright holder for this preprint (which was not certified by peer review) is the author/funder, who has granted medRxiv a license to display the preprint in perpetuity. This article is a US Government work. It is not subject to copyright under 17 USC 105 and is also made available for use under a CC0 license.

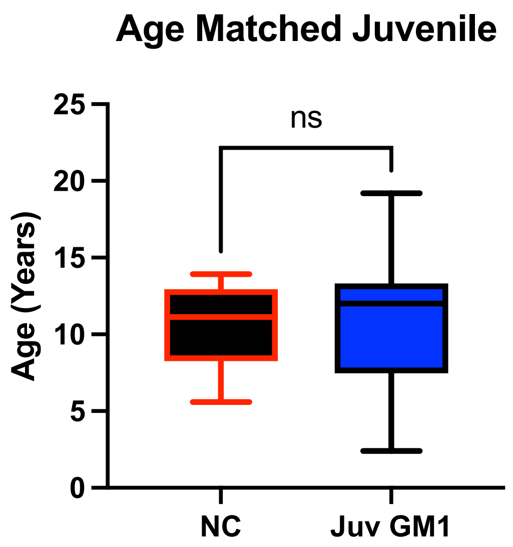

Figure C1. Age and sex-matched juvenile GM1 gangliosidosis cohort evaluated for differences by a 2 tailed *t*-test (t=0.1757, df =19, p = 0.8624).

medRxiv preprint doi: https://doi.org/10.1101/2025.02.18.25322304; this version posted February 20, 2025. The copyright holder for this preprint (which was not certified by peer review) is the author/funder, who has granted medRxiv a license to display the preprint in perpetuity.
This article is a US Government work. It is not subject to copyright under 17 USC 105 and is also made available for use under a CC0 license.

Supplement D: MRI Exclusion

Manual Segmentation Process
All 19 MRI scans from neurotypical controls, all 11 scans from 8 late-infantile GM1 gangliosidosis patients, and all 45 MRI scans from 16 juvenile GM1 gangliosidosis patients were analyzed using the manual segmentation method.

Freesurfer
All 19 MRI scans from 11 neurotypical controls were analyzed using the Freesurfer *recon-all* pipeline. 10 MRI scans from 7 late-infantile patients were analyzed using the Freesurfer *recon-all* pipeline, with one late-infantile patient MRI scan failing to process due to significant atrophy and demyelination (Figure D1). Thirty-nine MRI scans from 16 juvenile patients were analyzed using the Freesurfer *recon-all* pipeline with one 3 MRI scans failing to process due to the presence of bone marrow in the cerebrospinal fluid (Figure D2) and 3 MRI scans failing to process due to significant atrophy (Figure D3).

volBrain
All 19 MRI scans from 11 neurotypical controls were analyzed using volBrain's *vol2Brain* pipeline. Ten MRI scans from 7 late-infantile patients were analyzed using the volBrain's *vol2Brain* pipeline, with one late-infantile patient MRI scan failing to process due to significant atrophy and demyelination (Figure D1). All 45 MRI scans from 16 juvenile GM1 gangliosidosis patients were analyzed using the volBrain's *vol2Brain* segmentation method.

FSL
FSL FAST
All 19 MRI scans from 11 neurotypical controls were analyzed using FSL's *FAST* pipeline. Ten MRI scans from 7 late-infantile GM1 gangliosidosis patients were analyzed using FSL's *FAST* pipeline with one late-infantile patient MRI scan failing to process due to significant atrophy and demyelination (Figure D1). All 45 MRI scans from 16 juvenile GM1 gangliosidosis patients were analyzed using the FSL's *fast* segmentation method.

FSL FIRST
All 19 MRI scans from 11 neurotypical controls were analyzed using the FSL's *FIRST* pipeline. 10 MRI scans from 7 late-infantile patients were analyzed using FSL's *FIRST* with one late-infantile patient MRI scan failing to process due to significant atrophy (Figure D1). Forty-three MRI scans from 16 juvenile GM1 gangliosidosis patients were analyzed using the FSL *first* segmentation methods. Two MRI scans failed to process due to the presence of bone marrow in the cerebrospinal fluid (Figure D2) and significant atrophy (Figure D3, Scan3).

SPM12
All 19 MRI scans from 11 neurotypical controls were analyzed using the SPM12's *Segment* pipeline. All 11 MRI scans from late-infantile GM1 gangliosidosis patients were analyzed using SPM12's *Segment* pipeline. All 45 MRI scans from 16 juvenile GM1 gangliosidosis patients were analyzed using the SPM12's *Segment* segmentation method.

medRxiv preprint doi: https://doi.org/10.1101/2025.02.18.25322304; this version posted February 20, 2025. The copyright holder for this preprint (which was not certified by peer review) is the author/funder, who has granted medRxiv a license to display the preprint in perpetuity. This article is a US Government work. It is not subject to copyright under 17 USC 105 and is also made available for use under a CC0 license.

SimNIBS' Headreco

All 19 MRI scans from 11 neurotypical controls were analyzed using the SimNIBS' *Headreco* pipeline. Ten MRI scans from 7 late-infantile patients were analyzed using the SimNIBS' *Headreco* pipeline, with one late-infantile patient MRI scan failing to process due to significant atrophy and demyelination (Figure D1). Forty-three scans from 16 juvenile GM1 patients were analyzed using SimNIBS. Two scans from a juvenile participant of the GM1 gangliosidosis cohort were excluded from this analysis due to the presence of bone marrow in the cerebrospinal fluid (Figure D2).

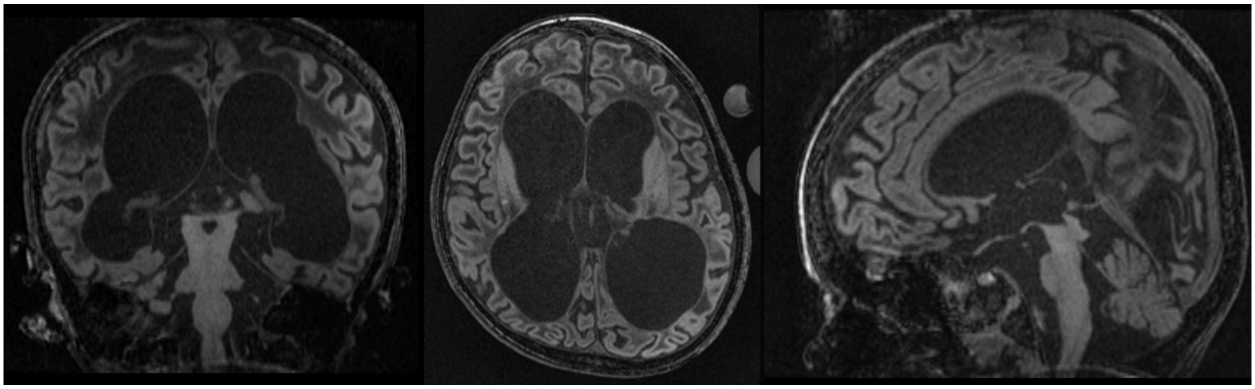

Figure D1. Coronal, Axial, and Sagittal T1-weighted MRI of a late-infantile GM1 patient with significant cerebral atrophy, ventricle enlargement, and hypomyelination.

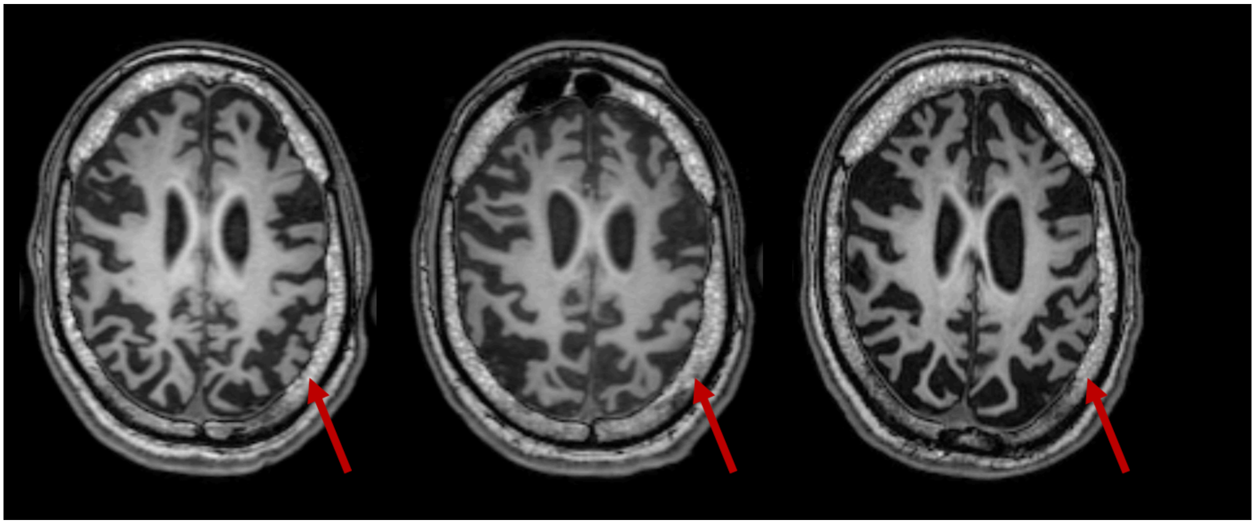

Figure D2. Serial T1 MRI of an excluded juvenile GM1 patient at age 11-15 (Scan 1), 16-20 (Scan 2), and 16-20 (Scan 3) years old with bone marrow (red arrow) in the cerebrospinal fluid.

medRxiv preprint doi: https://doi.org/10.1101/2025.02.18.25322304; this version posted February 20, 2025. The copyright holder for this preprint (which was not certified by peer review) is the author/funder, who has granted medRxiv a license to display the preprint in perpetuity.
This article is a US Government work. It is not subject to copyright under 17 USC 105 and is also made available for use under a CC0 license.

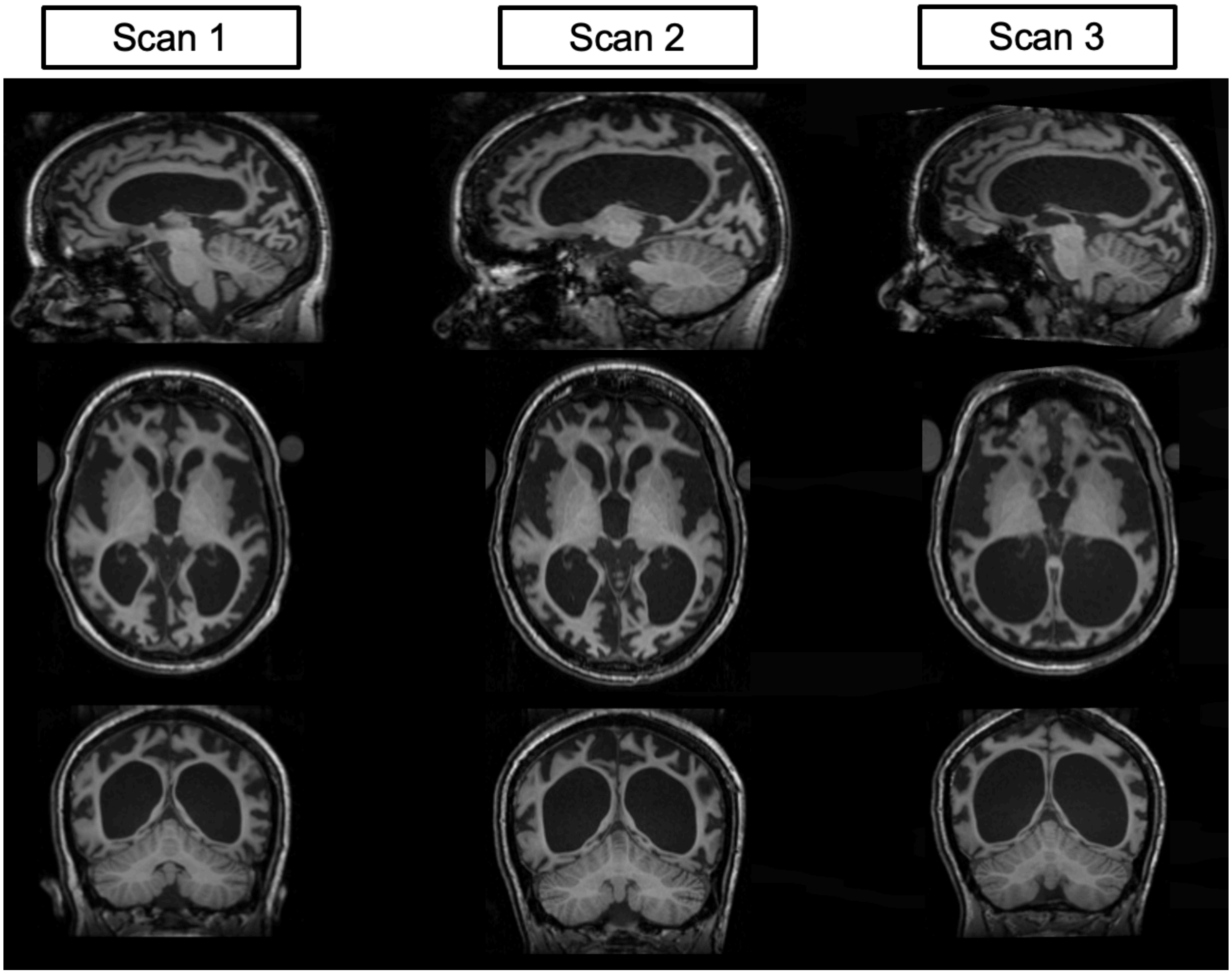

Figure D3. Serial T1 MRI imaging of a juvenile GM1 patient at age 16-20 (Scan 1), 16-20 (Scan 2), and 21-25 (Scan 3) years old with significant atrophy and ventricle enlargement across three evaluations.

medRxiv preprint doi: https://doi.org/10.1101/2025.02.18.25322304; this version posted February 20, 2025. The copyright holder for this preprint (which was not certified by peer review) is the author/funder, who has granted medRxiv a license to display the preprint in perpetuity.
This article is a US Government work. It is not subject to copyright under 17 USC 105 and is also made available for use under a CC0 license.

Supplementary Results

Supplement E. Cross-sectional Analysis of Late-Infantile Patients

Cerebellum

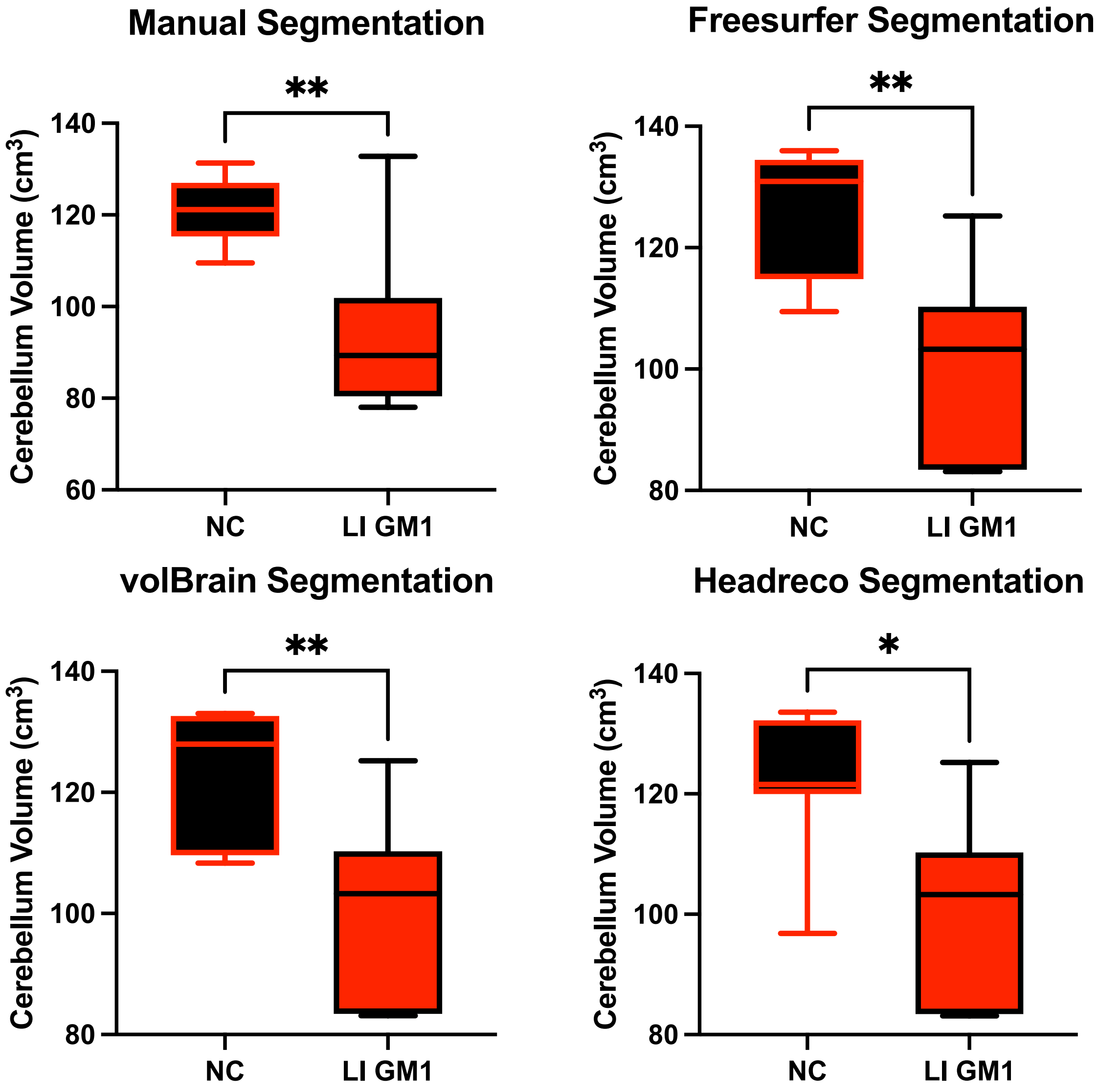

Figure E1. Cross-sectional evaluation of the 3 automated segmentation algorithms to demonstrate cohort differences in cerebellar volume. Late-infantile (LI) GM1 patients are shown in Red. Neurotypical controls (NC) are shown in black. *P*-values were calculated from the *t*-statistic. * $P < 0.05$, ** $P < 0.01$, *** $P < 0.001$, **** $P < 0.0001$.

medRxiv preprint doi: https://doi.org/10.1101/2025.02.18.25322304; this version posted February 20, 2025. The copyright holder for this preprint (which was not certified by peer review) is the author/funder, who has granted medRxiv a license to display the preprint in perpetuity. This article is a US Government work. It is not subject to copyright under 17 USC 105 and is also made available for use under a CC0 license.

Thalamus

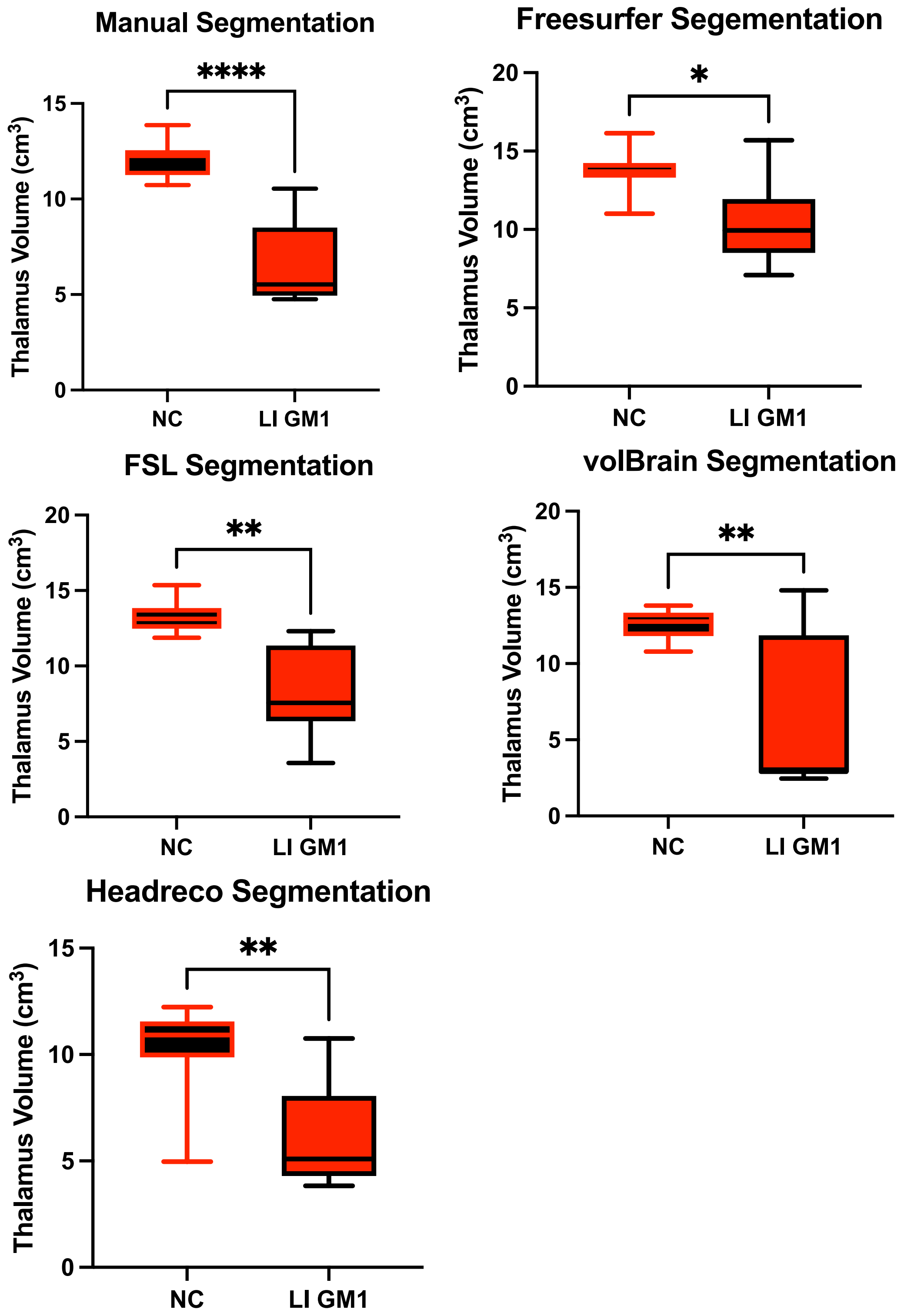

Figure E2. Cross-sectional evaluation of the 4 automated segmentation algorithms to demonstrate cohort differences in thalamic volume. Late-infantile (LI) GM1 patients are shown in Red. Neurotypical controls (NC) are shown in black. *P*-values were calculated from the *t*-statistic. * $P < 0.05$, ** $P < 0.01$, *** $P < 0.001$, **** $P < 0.0001$.

medRxiv preprint doi: https://doi.org/10.1101/2025.02.18.25322304; this version posted February 20, 2025. The copyright holder for this preprint (which was not certified by peer review) is the author/funder, who has granted medRxiv a license to display the preprint in perpetuity.
This article is a US Government work. It is not subject to copyright under 17 USC 105 and is also made available for use under a CC0 license.

Caudate Nucleus

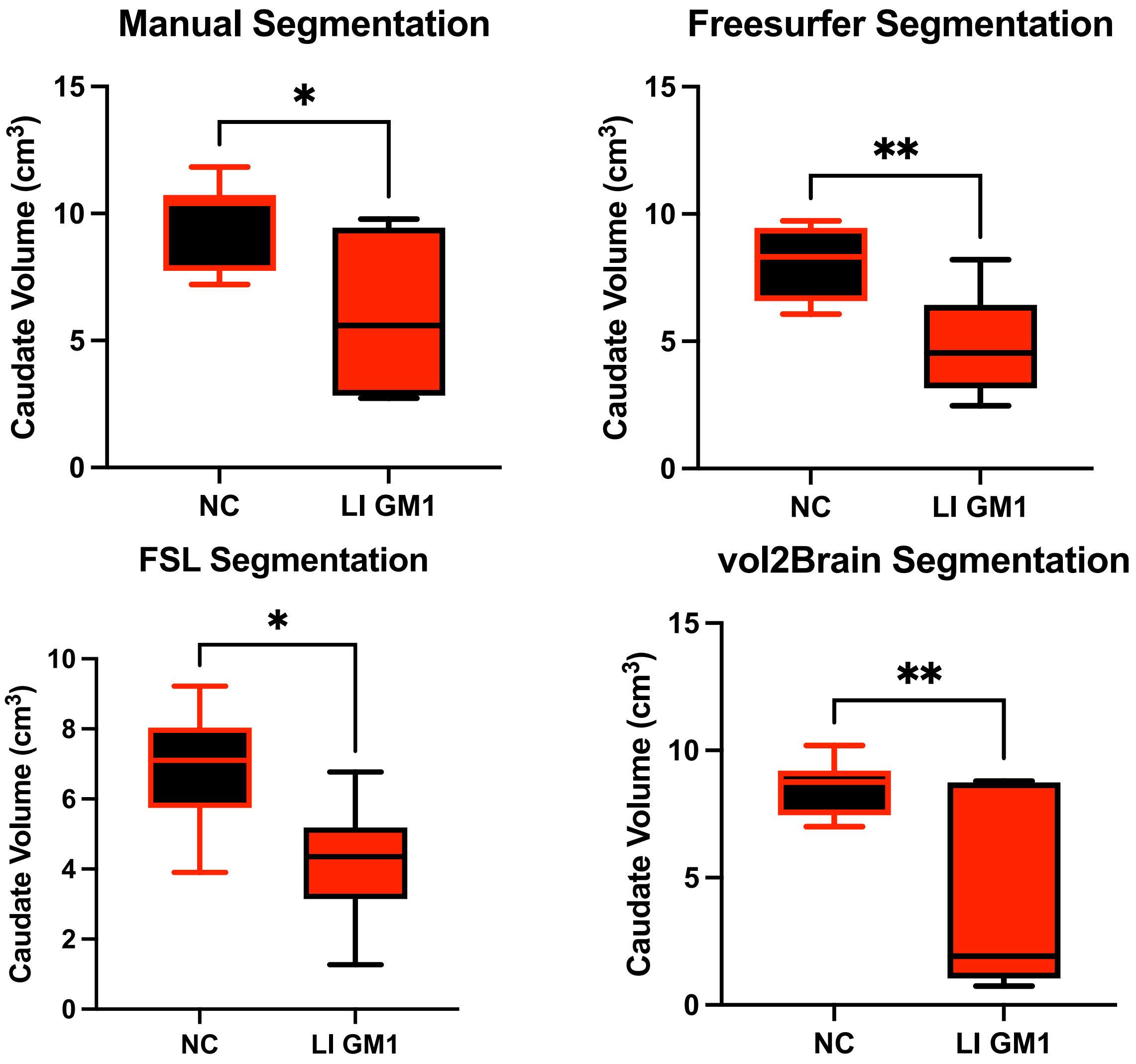

Figure E3. Cross-sectional evaluation of the 3 automated segmentation algorithms to demonstrate cohort differences in caudate volume. Late-infantile (LI) GM1 patients are shown in Red. Neurotypical controls (NC) are shown in black. *P*-values were calculated from the *t*-statistic. * $P < 0.05$, ** $P < 0.01$, *** $P < 0.001$, **** $P < 0.0001$.

medRxiv preprint doi: https://doi.org/10.1101/2025.02.18.25322304; this version posted February 20, 2025. The copyright holder for this preprint (which was not certified by peer review) is the author/funder, who has granted medRxiv a license to display the preprint in perpetuity. This article is a US Government work. It is not subject to copyright under 17 USC 105 and is also made available for use under a CC0 license.

Lentiform Nucleus

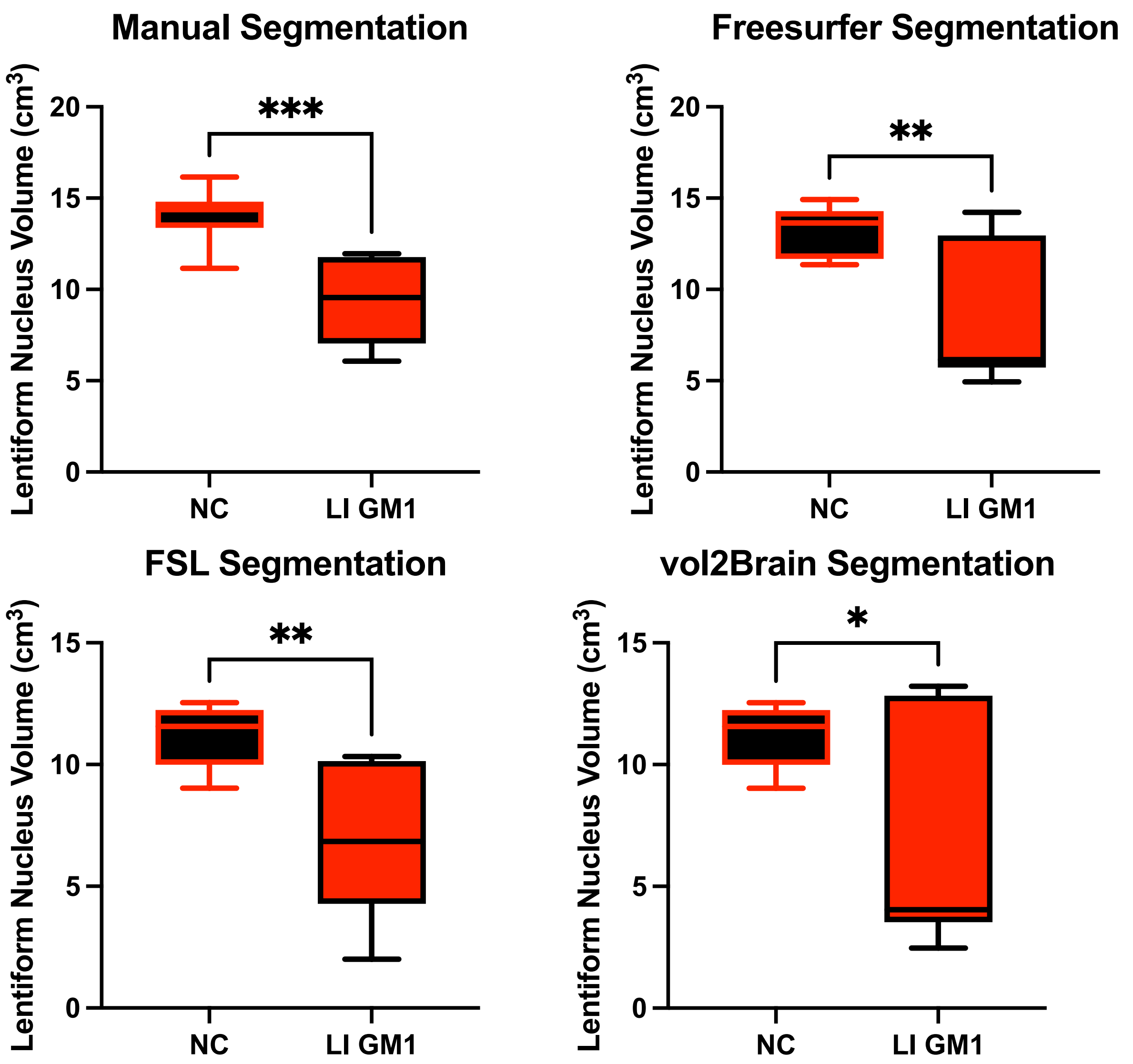

Figure E4. Cross-sectional evaluation of the 3 automated segmentation algorithms to demonstrate cohort differences in lentiform nucleus volume. Late-infantile (LI) GM1 patients are shown in Red. Neurotypical controls (NC) are shown in black. P-values were calculated from the t-statistic. * $P < 0.05$, ** $P < 0.01$, *** $P < 0.001$, **** $P < 0.0001$.

medRxiv preprint doi: https://doi.org/10.1101/2025.02.18.25322304; this version posted February 20, 2025. The copyright holder for this preprint (which was not certified by peer review) is the author/funder, who has granted medRxiv a license to display the preprint in perpetuity.
This article is a US Government work. It is not subject to copyright under 17 USC 105 and is also made available for use under a CC0 license.

Corpus Callosum

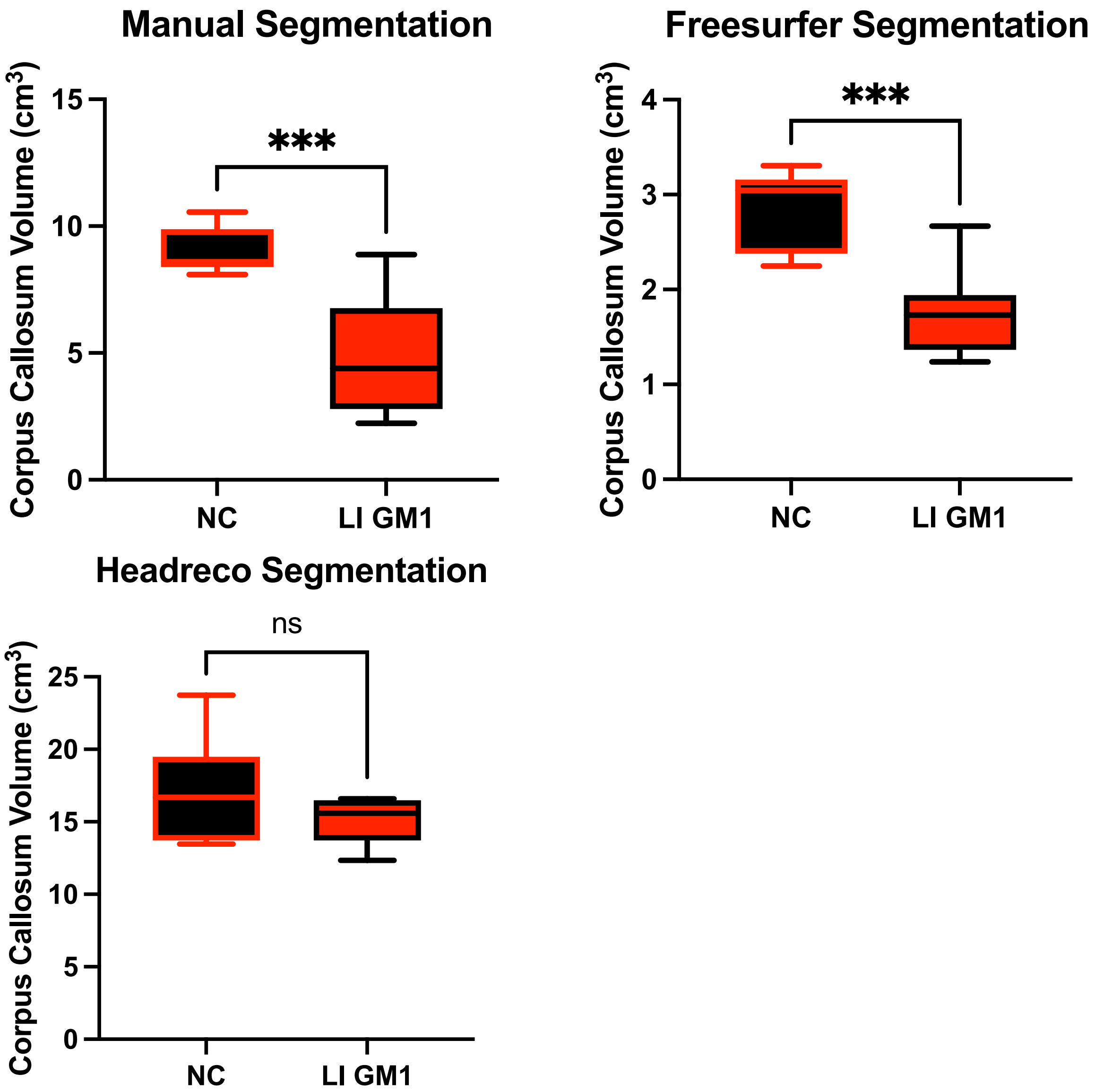

Figure E5. Cross-sectional evaluation of the 2 automated segmentation algorithms to demonstrate cohort differences in corpus callosum volume. Late-infantile (LI) GM1 patients are shown in Red. Neurotypical controls (NC) are shown in black. P-values were calculated from the t-statistic. * $P < 0.05$, ** $P < 0.01$, *** $P < 0.001$, **** $P < 0.0001$.

medRxiv preprint doi: https://doi.org/10.1101/2025.02.18.25322304; this version posted February 20, 2025. The copyright holder for this preprint
(which was not certified by peer review) is the author/funder, who has granted medRxiv a license to display the preprint in perpetuity.
This article is a US Government work. It is not subject to copyright under 17 USC 105 and is also made available
for use under a CC0 license.

Supplement F. Cross-sectional Analysis of Juvenile Patients

Ventricles

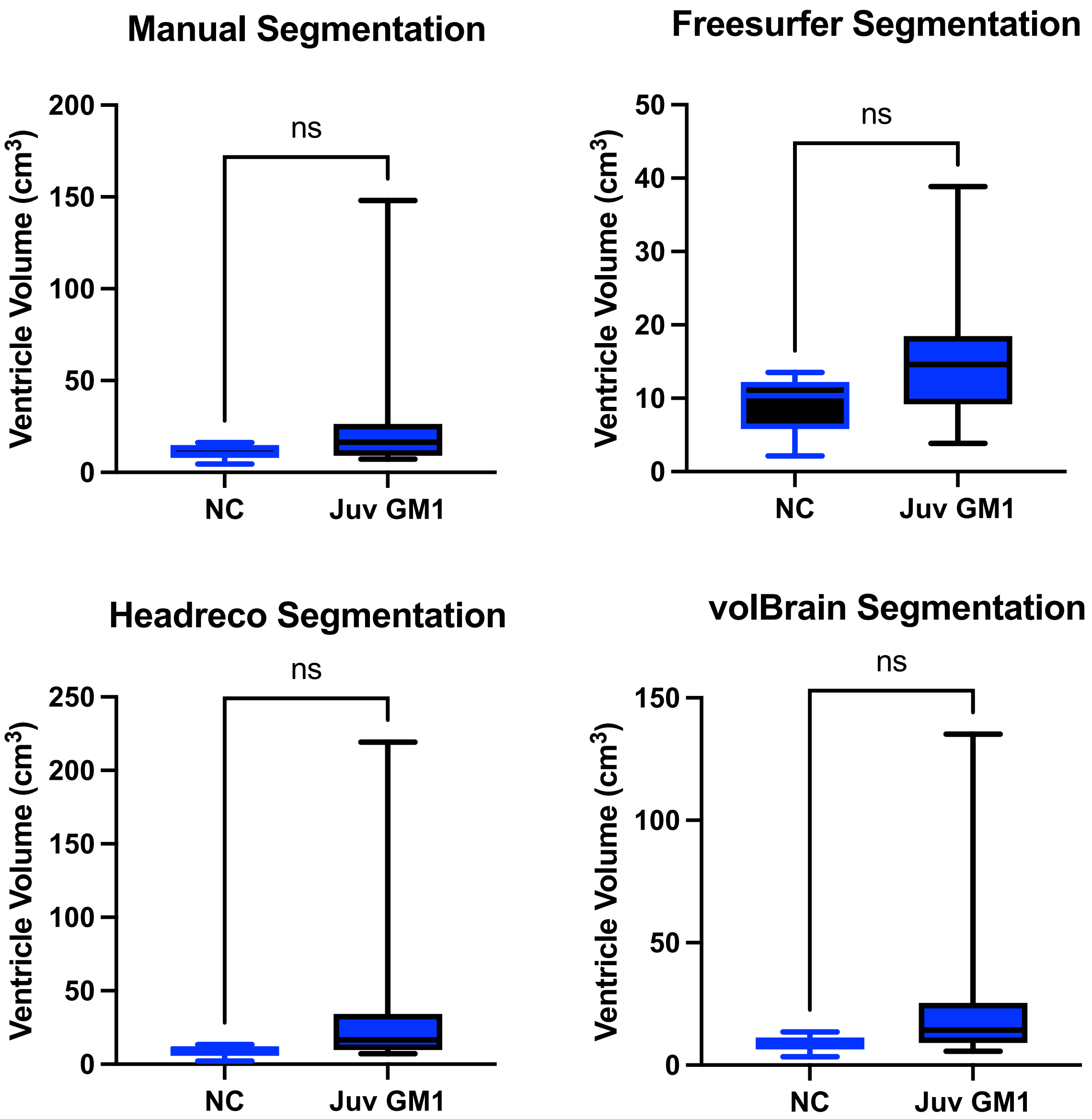

Figure F1. Cross-sectional evaluation of the 3 automated segmentation algorithms to demonstrate cohort differences in ventricle volume. Juvenile (Juv) GM1 patients are shown in blue. Neurotypical controls (NC) are shown in black. P-values were calculated from the t-statistic. * $P < 0.05$, ** $P < 0.01$, *** $P < 0.001$, **** $P < 0.0001$.

medRxiv preprint doi: https://doi.org/10.1101/2025.02.18.25322304; this version posted February 20, 2025. The copyright holder for this preprint (which was not certified by peer review) is the author/funder, who has granted medRxiv a license to display the preprint in perpetuity. This article is a US Government work. It is not subject to copyright under 17 USC 105 and is also made available for use under a CC0 license.

Cerebellum

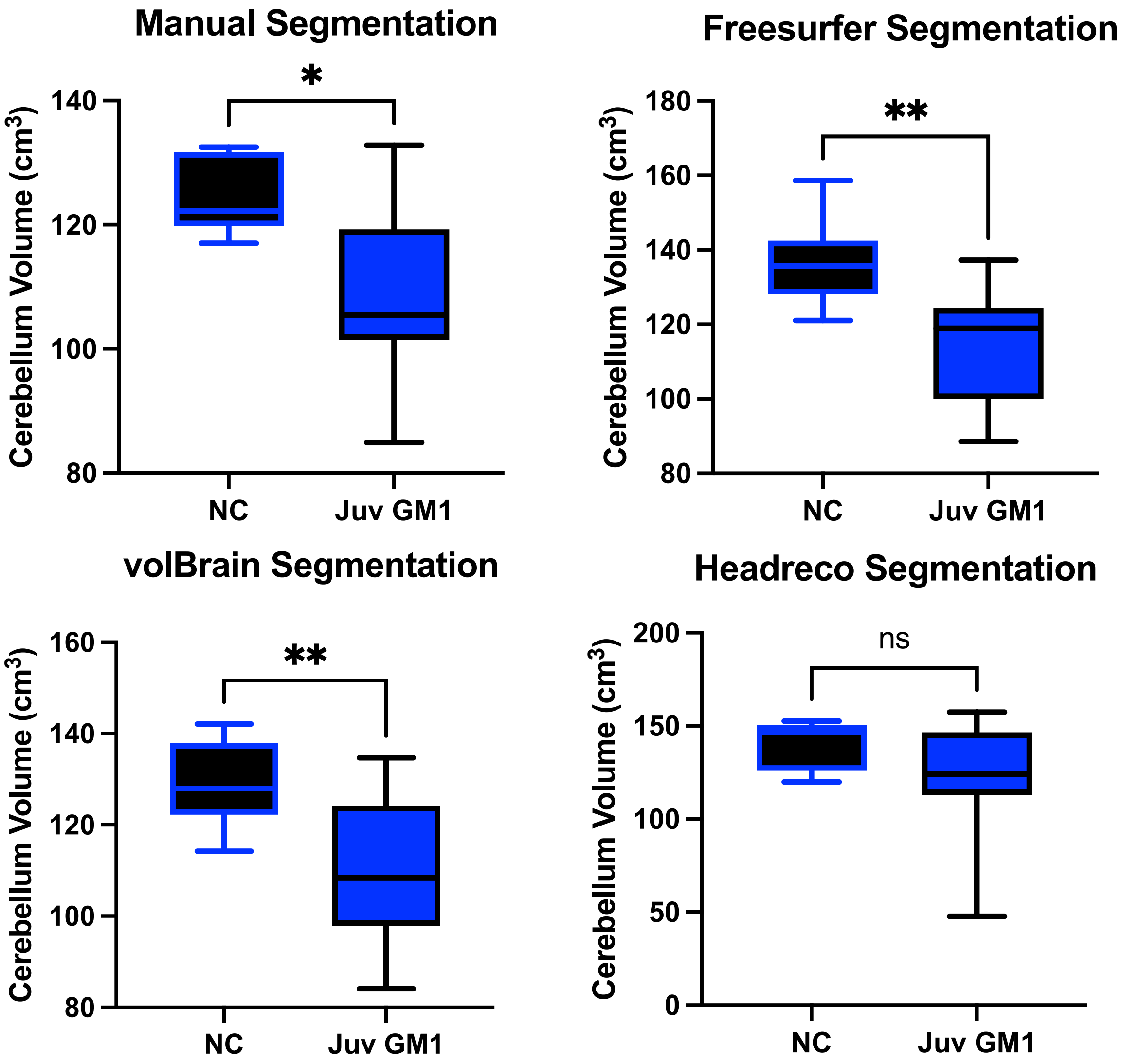

Figure F2. Cross-sectional evaluation of the 3 automated segmentation algorithms to demonstrate cohort differences in cerebellum volume. Juvenile (Juv) GM1 patients are shown in blue. Neurotypical controls (NC) are shown in black. *P*-values were calculated from the *t*-statistic. * $P < 0.05$, ** $P < 0.01$, *** $P < 0.001$, **** $P < 0.0001$.

medRxiv preprint doi: https://doi.org/10.1101/2025.02.18.25322304; this version posted February 20, 2025. The copyright holder for this preprint
(which was not certified by peer review) is the author/funder, who has granted medRxiv a license to display the preprint in perpetuity.
This article is a US Government work. It is not subject to copyright under 17 USC 105 and is also made available
for use under a CC0 license.

Caudate Nucleus

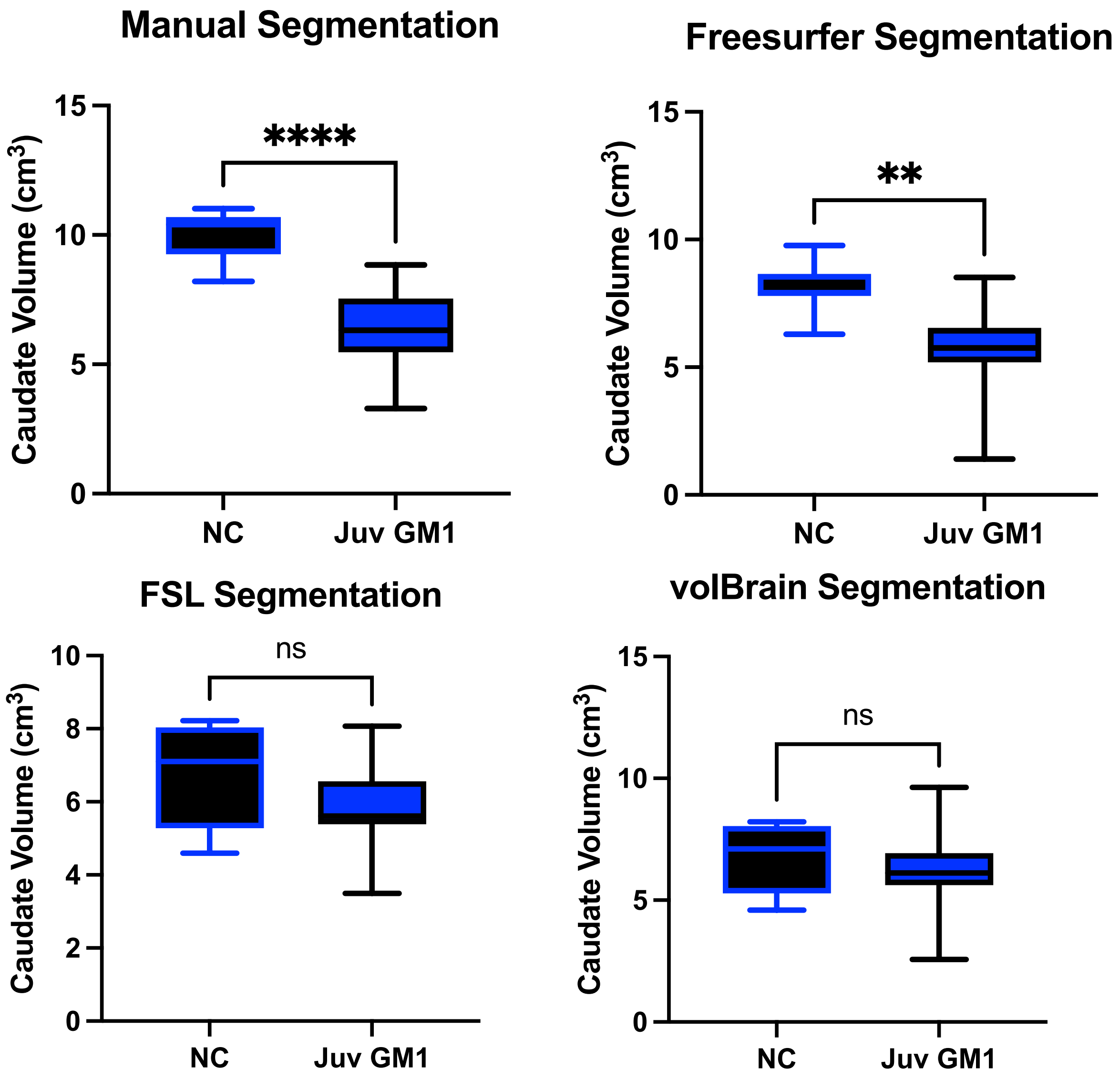

Figure F3. Cross-sectional evaluation of the 3 automated segmentation algorithms to demonstrate cohort differences in caudate volume. Juvenile (Juv) GM1 patients are shown in blue. Neurotypical controls (NC) are shown in black. *P*-values were calculated from the *t*-statistic. * $P < 0.05$, ** $P < 0.01$, *** $P < 0.001$, **** $P < 0.0001$.

medRxiv preprint doi: https://doi.org/10.1101/2025.02.18.25322304; this version posted February 20, 2025. The copyright holder for this preprint
(which was not certified by peer review) is the author/funder, who has granted medRxiv a license to display the preprint in perpetuity.
This article is a US Government work. It is not subject to copyright under 17 USC 105 and is also made available
for use under a CC0 license.

Lentiform Nucleus

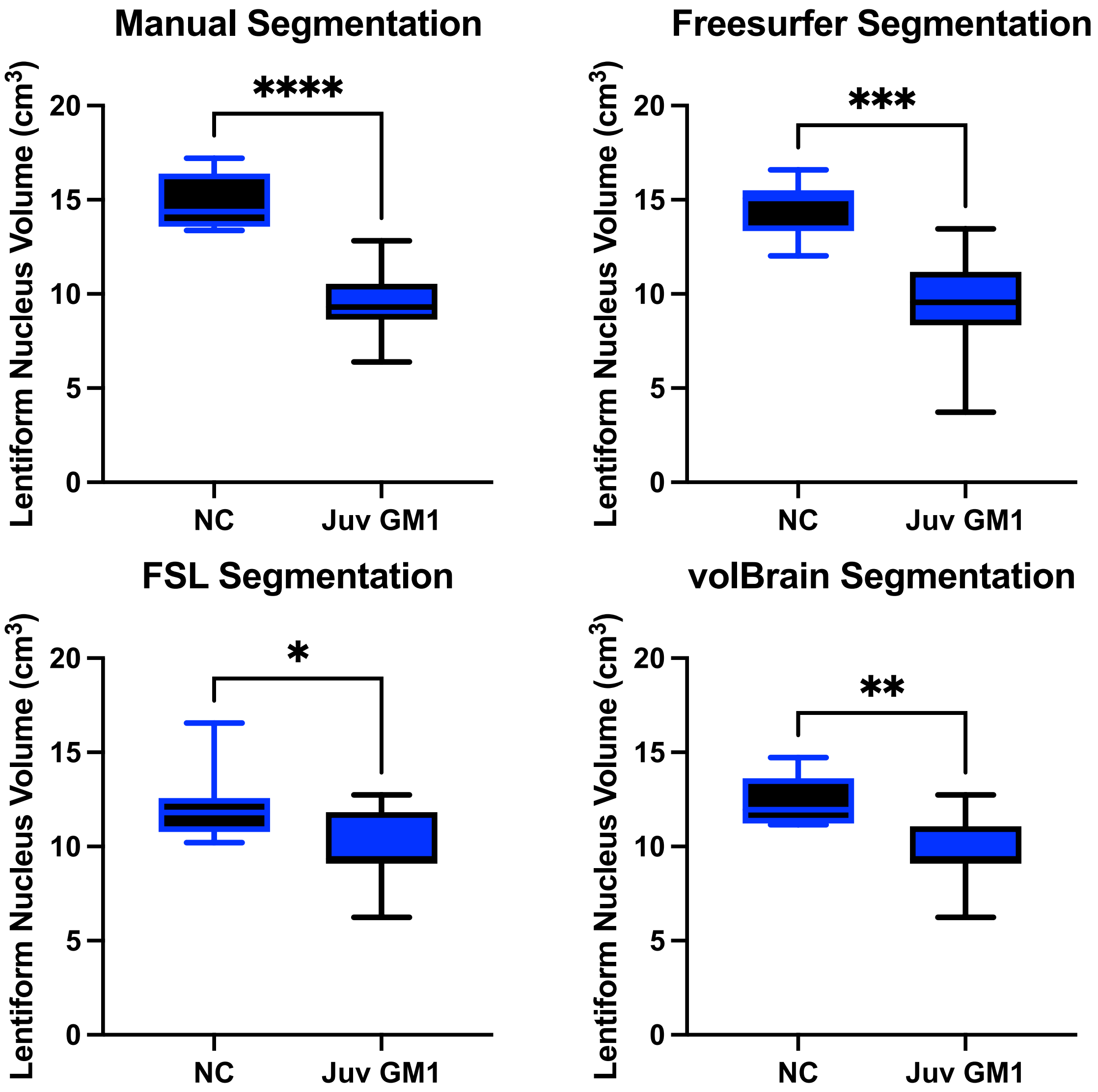

Figure F4. Cross-sectional evaluation of the 3 automated segmentation algorithms to demonstrate cohort differences in lentiform nucleus volume. Juvenile (Juv) GM1 patients are shown in blue. Neurotypical controls (NC) are shown in black. *P*-values were calculated from the *t*-statistic. * $P < 0.05$, ** $P < 0.01$, *** $P < 0.001$, **** $P < 0.0001$.

medRxiv preprint doi: https://doi.org/10.1101/2025.02.18.25322304; this version posted February 20, 2025. The copyright holder for this preprint
(which was not certified by peer review) is the author/funder, who has granted medRxiv a license to display the preprint in perpetuity.
This article is a US Government work. It is not subject to copyright under 17 USC 105 and is also made available
for use under a CC0 license.

Corpus Callosum

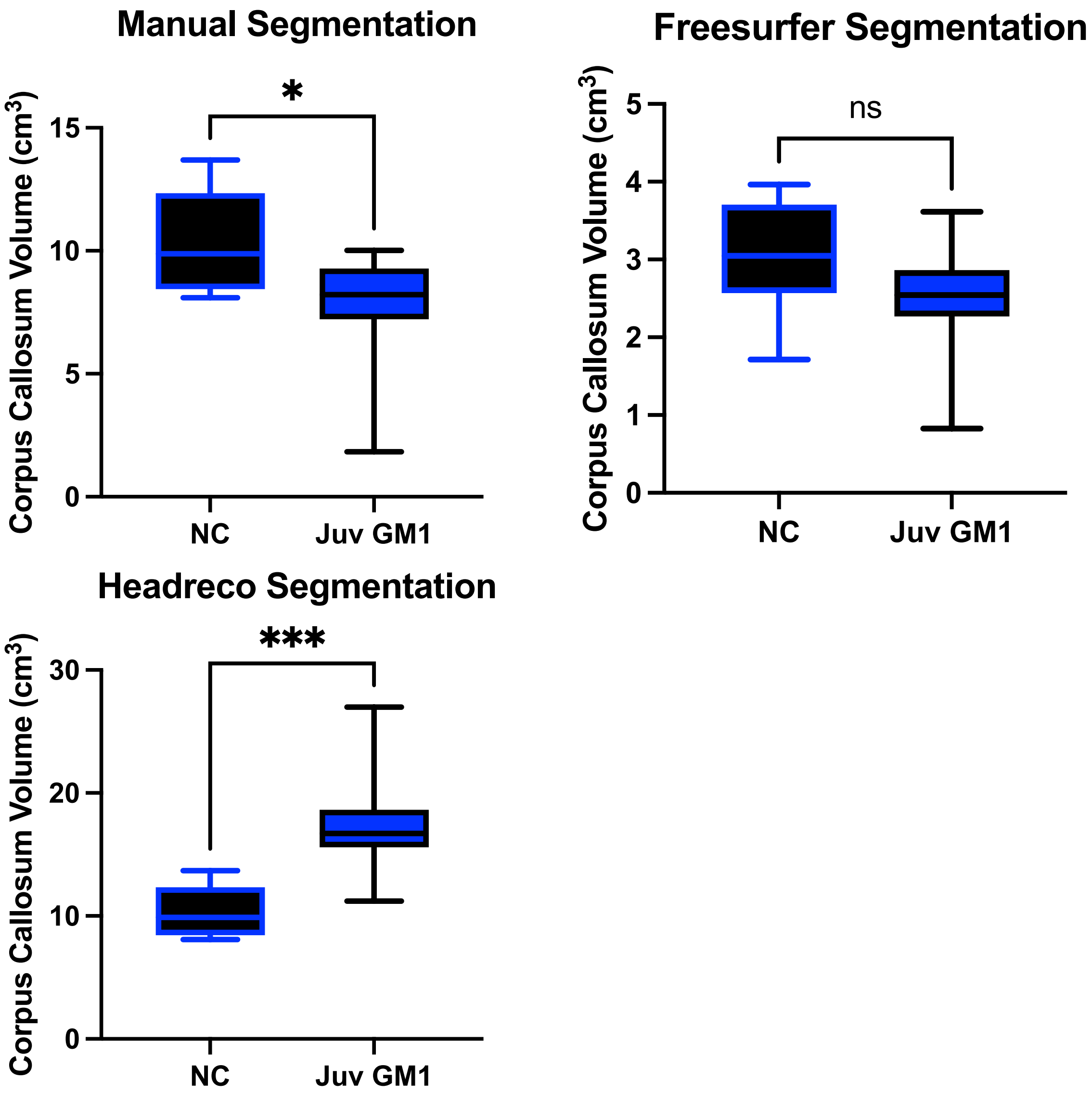

Figure F5. Cross-sectional evaluation of the 2 automated segmentation algorithms to demonstrate cohort differences in corpus callosum volume. Juvenile (Juv) GM1 patients are shown in blue. Neurotypical controls (NC) are shown in black. *P*-values were calculated from the *t*-statistic. * $P < 0.05$, ** $P < 0.01$, *** $P < 0.001$, **** $P < 0.0001$.

medRxiv preprint doi: https://doi.org/10.1101/2025.02.18.25322304; this version posted February 20, 2025. The copyright holder for this preprint (which was not certified by peer review) is the author/funder, who has granted medRxiv a license to display the preprint in perpetuity.
This article is a US Government work. It is not subject to copyright under 17 USC 105 and is also made available for use under a CC0 license.

Supplement G: Correlation Strength Tables between Manual and Automated Segmentation

Table GI. Comparison of correlation coefficients (R^2, top row) between the 5 fully automated segmentation pipelines and the manual pipeline in the 7 different brain regions for the neurotypical controls. Normality was determined using the Shapiro-Wilk test. *P*-values (bottom row) were calculated from the Pearson product moment correlation coefficient when the data was normally distributed and from Spearman's rank correlation coefficient when the data was not normally distributed and designated with a $^+$. N/A are designations where the region was not calculated using the specified segmentation algorithm.

Structure	Freesurfer	FSL	volBrain	SPM	SimNIBS Headreco (SPM+CAT)
**Whole Brain**	0.8256<br>$p < 0.0001$	0.9058<br>$p < 0.0001$	0.9852<br>$p < 0.0001$	0.8629<br>$p < 0.0001^+$	0.8593<br>$p < 0.0001$
**Ventricles**	0.6180<br>$p < 0.0001$	N/A	0.9452<br>$p < 0.0001$	N/A	0.7951<br>$p < 0.0001$
**Cerebellum**	0.6910<br>$p < 0.0001$	N/A	0.8269<br>$p < 0.0001$	N/A	0.5384<br>$p = 0.0003$
**Thalamus**	0.5359<br>$p = 0.0004$	0.5074<br>$p = 0.0006$	0.6276<br>$p < 0.0001$	N/A	0.5322<br>$p < 0.0001^+$
**Caudate**	0.8174<br>$p < 0.0001$	0.5416<br>$p = 0.0003$	0.7282<br>$p < 0.0001$	N/A	N/A
**Lentiform Nucleus**	0.7251<br>$p < 0.0001$	0.5271<br>$p = 0.0004^+$	0.5562<br>$p = 0.0002$	N/A	N/A
**Corpus Callosum**	0.06547<br>$p = 0.2904$	N/A	N/A	N/A	0.4829<br>$p = 0.0010$

medRxiv preprint doi: https://doi.org/10.1101/2025.02.18.25322304; this version posted February 20, 2025. The copyright holder for this preprint (which was not certified by peer review) is the author/funder, who has granted medRxiv a license to display the preprint in perpetuity. This article is a US Government work. It is not subject to copyright under 17 USC 105 and is also made available for use under a CC0 license.

Table GII. Comparison of correlation coefficients (R^2, top row) between the 5 fully automated segmentation pipelines and the manual pipeline in the 7 different brain regions for the juvenile GM1 patients. Normality was determined using the Shapiro-Wilk test. *P*-values (bottom row) were calculated from the Pearson product moment correlation coefficient when the data was normally distributed and from Spearman's rank correlation coefficient when the data was not normally distributed and designated with a $^+$. N/A are designations where the region was not calculated using the specified segmentation algorithm.

Structure	Freesurfer	FSL	volBrain	SPM	SimNIBS Headreco (SPM+CAT)
**Whole Brain**	0.9196<br>$p < 0.0001^+$	0.6038<br>$p < 0.0001$	0.9583<br>$p < 0.0001^+$	0.9642<br>$p < 0.0001^+$	0.8956<br>$p < 0.0001^+$
**Ventricles**	0.9765<br>$p < 0.0001^+$	N/A	0.9782<br>$p < 0.0001^+$	N/A	0.7422<br>$p < 0.0001^+$
**Cerebellum**	0.9133<br>$p < 0.0001$	N/A	0.9258<br>$p < 0.0001$	N/A	0.6009<br>$p < 0.0001^+$
**Thalamus**	0.4359<br>$p < 0.0001$	0.5033<br>$p < 0.0001^+$	0.8053<br>$p < 0.0001^+$	N/A	0.7073<br>$p < 0.0001^+$
**Caudate**	0.8181<br>$p < 0.0001^+$	0.5068<br>$p < 0.0001^+$	0.8885<br>$p < 0.0001^+$	N/A	N/A
**Lentiform Nucleus**	0.5660<br>$p < 0.0001$	0.4266<br>$p < 0.0001$	0.6605<br>$p < 0.0001$	N/A	N/A
**Corpus Callosum**	0.4050<br>$p = 0.0122^+$	N/A	N/A	N/A	0.3131<br>$p < 0.0001$

medRxiv preprint doi: https://doi.org/10.1101/2025.02.18.25322304; this version posted February 20, 2025. The copyright holder for this preprint (which was not certified by peer review) is the author/funder, who has granted medRxiv a license to display the preprint in perpetuity. This article is a US Government work. It is not subject to copyright under 17 USC 105 and is also made available for use under a CC0 license.

Table GIII. Comparison of correlation coefficients (R^2, top row) between the 5 fully automated segmentation pipelines and the manual pipeline in the 7 different brain regions for the late-infantile GM1 patients. Normality was determined using the Shapiro-Wilk test. *P*-values (bottom row) were calculated from the Pearson product moment correlation coefficient when the data was normally distributed and from Spearman's rank correlation coefficient when the data was not normally distributed and designated with a $^+$. N/A are designations where the region was not calculated using the specified segmentation algorithm.

Structure	Freesurfer	FSL	volBrain	SPM	SimNIBS Headreco (SPM+CAT)
**Whole Brain**	0.3460<br>$p = 0.0736$	0.6585<br>$p = 0.0044$	0.9169<br>$p = 0.0072^+$	0.5458<br>$p = 0.0094$	0.8246<br>$p = 0.0003$
**Ventricles**	0.7467<br>$p = 0.0013$	N/A	0.9974<br>$p < 0.0001$	N/A	0.9950<br>$p < 0.0001$
**Cerebellum**	0.6728<br>$p = 0.0037$	N/A	0.8374<br>$p < 0.0001$	N/A	0.8779<br>$p < 0.0001$
**Thalamus**	0.5347<br>$p = 0.0163$	0.6585<br>$p = 0.0044$	0.8632<br>$p < 0.0001^+$	N/A	0.7809<br>$p = 0.0003$
**Caudate**	0.8536<br>$p = 0.0001$	0.2584<br>$p = 0.1335$	0.7394<br>$p = 0.0027^+$	N/A	N/A
**Lentiform Nucleus**	0.6619<br>$p = 0.0347^+$	0.7713<br>$p = 0.0008$	0.6518<br>$p = 0.0368^+$	N/A	N/A
**Corpus Callosum**	0.06030<br>$p = 0.4941$	N/A	N/A	N/A	< 0.01<br>$p = 0.9895^+$

medRxiv preprint doi: https://doi.org/10.1101/2025.02.18.25322304; this version posted February 20, 2025. The copyright holder for this preprint (which was not certified by peer review) is the author/funder, who has granted medRxiv a license to display the preprint in perpetuity.
This article is a US Government work. It is not subject to copyright under 17 USC 105 and is also made available for use under a CC0 license.

Supplement H: Correlations between Manual and Freesurfer Segmentation

Neurotypical Controls

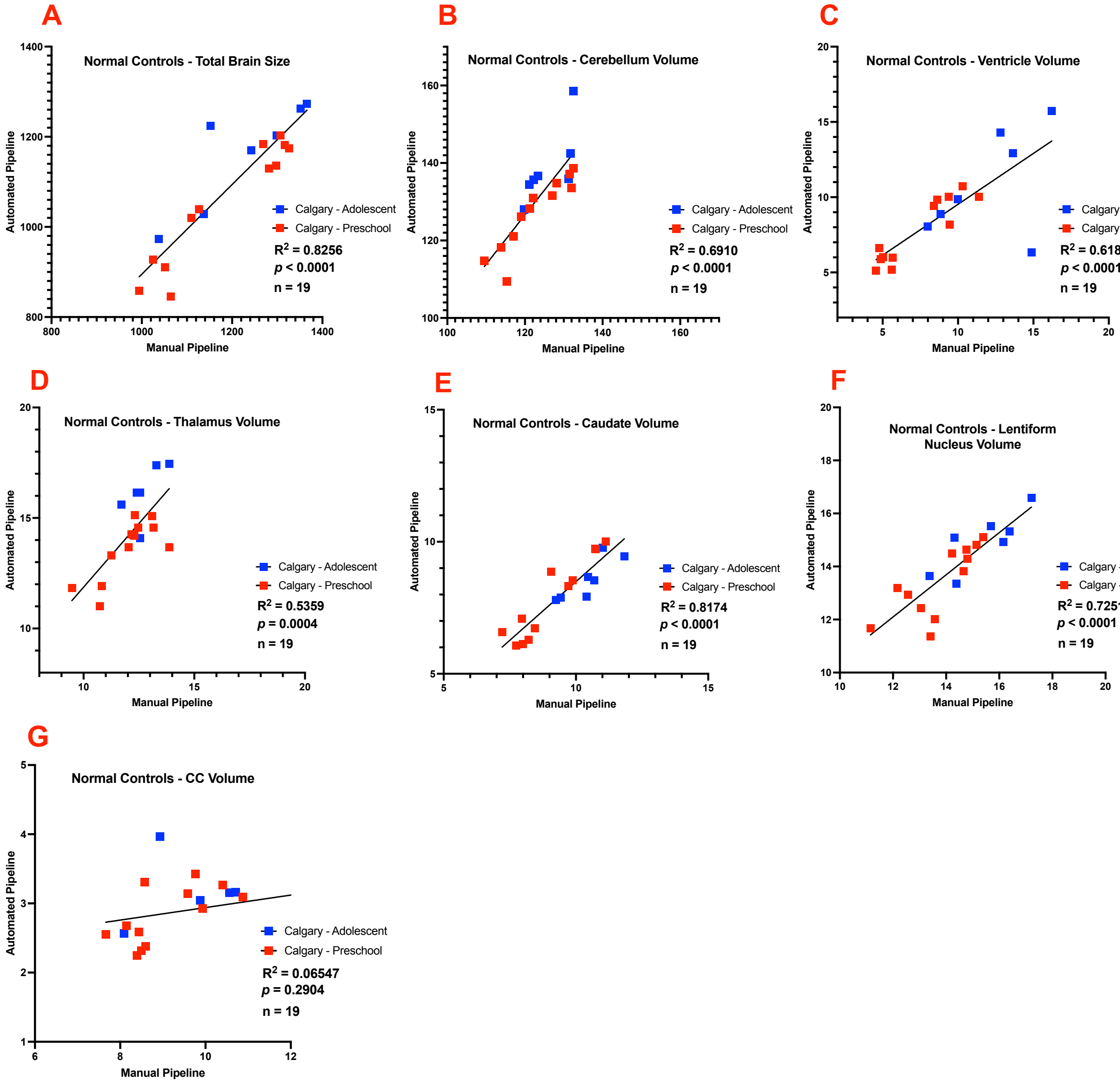

Figure H1. Correlations between Freesurfer's automated and the manual approach in neurotypical controls for the A.) Total brain volume B.) Cerebellum volume C.) Ventricle volume D.) Thalamic volume E.) Caduate volume F.) Lentiform nucleus volume and G.) Corpus callosum volume. Participants from the Calgary preschool MRI data set are shown in red and participants from the adolescent Calgary dataset are shown in blue. All volumes are shown in cm^3.

medRxiv preprint doi: https://doi.org/10.1101/2025.02.18.25322304; this version posted February 20, 2025. The copyright holder for this preprint (which was not certified by peer review) is the author/funder, who has granted medRxiv a license to display the preprint in perpetuity. This article is a US Government work. It is not subject to copyright under 17 USC 105 and is also made available for use under a CC0 license.

Juvenile GM1 Patients

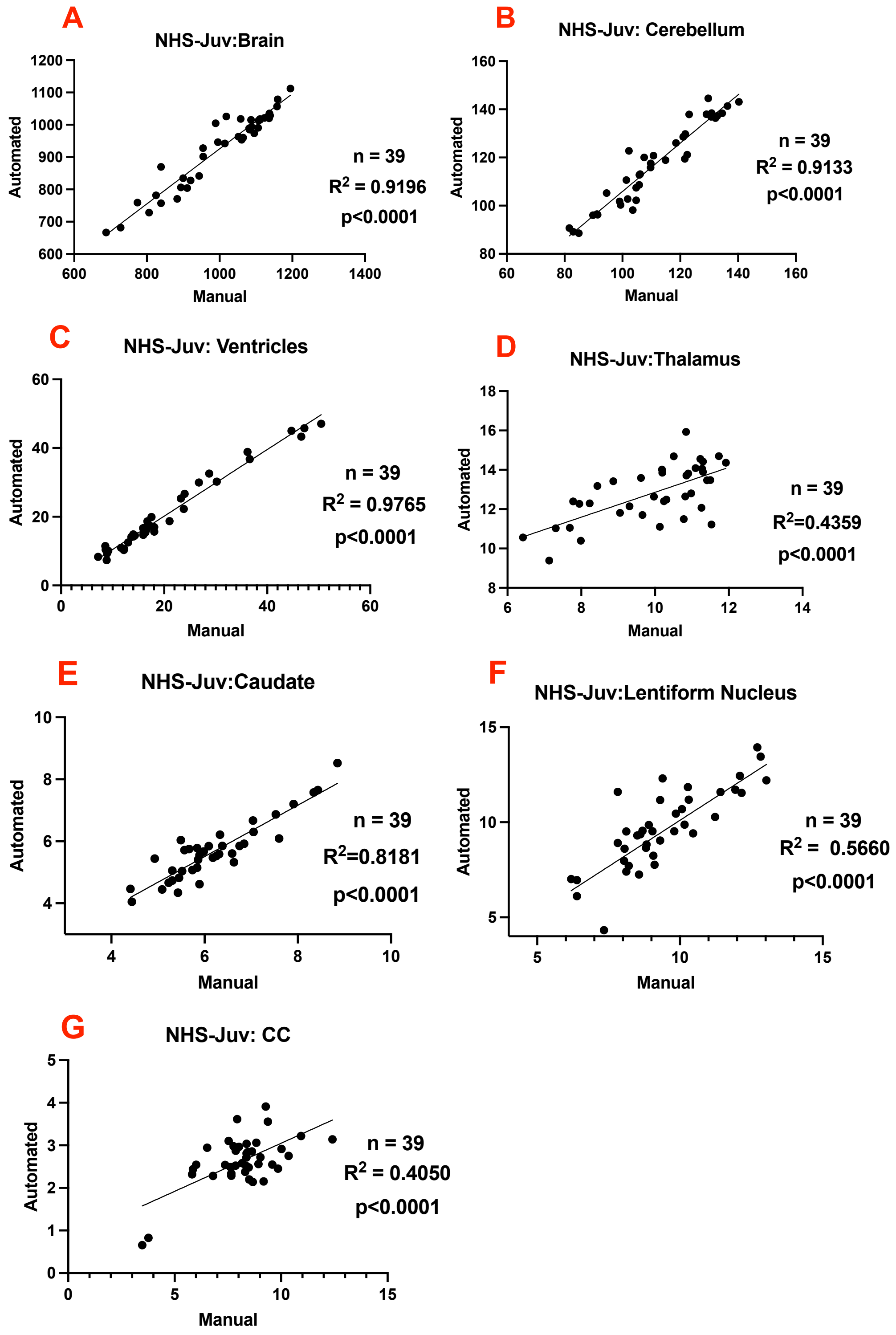

Figure H2. Correlations between Freesurfer's automated and the manual approach in natural history study (NHS) juvenile GM1 gangliosidosis patients for the A.) Total brain volume B.) Cerebellum volume C.) Ventricle volume D.) Thalamic volume E.) Caduate volume F.) Lentiform nucleus volume and G.) Corpus callosum volume. All volumes are shown in cm^3.

medRxiv preprint doi: https://doi.org/10.1101/2025.02.18.25322304; this version posted February 20, 2025. The copyright holder for this preprint
(which was not certified by peer review) is the author/funder, who has granted medRxiv a license to display the preprint in perpetuity.
This article is a US Government work. It is not subject to copyright under 17 USC 105 and is also made available
for use under a CC0 license.

Late-infantile GM1 Patients

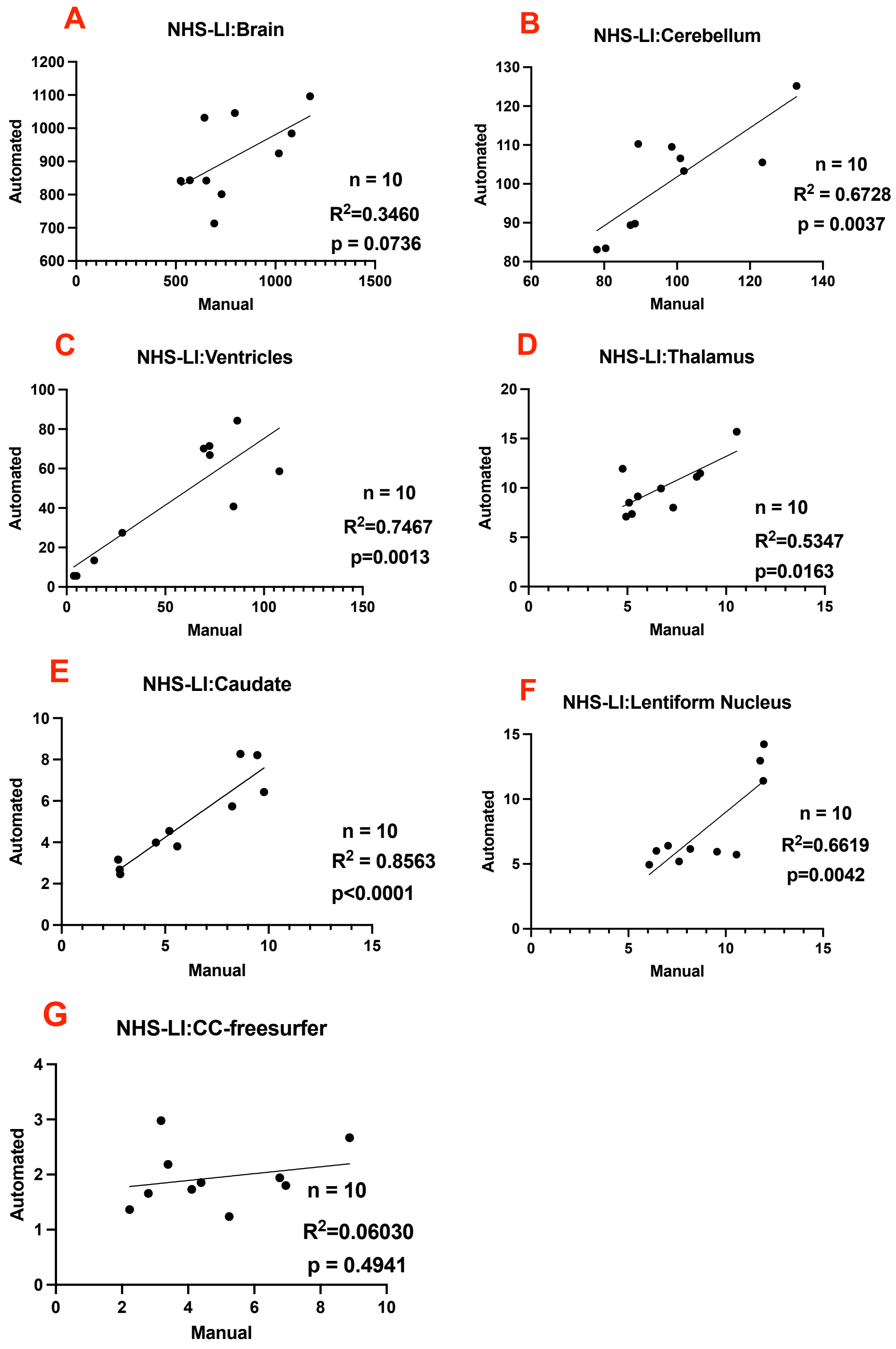

Figure H3. Correlations between Freesurfer's automated and the manual approach in natural history study (NHS) late-infantile GM1 gangliosidosis patients for the A.) Total brain volume B.) Cerebellum volume C.) Ventricle volume D.) Thalamic volume E.) Caduate volume F.) Lentiform nucleus volume and G.) Corpus callosum volume. All volumes are shown in cm^3.

medRxiv preprint doi: https://doi.org/10.1101/2025.02.18.25322304; this version posted February 20, 2025. The copyright holder for this preprint (which was not certified by peer review) is the author/funder, who has granted medRxiv a license to display the preprint in perpetuity. This article is a US Government work. It is not subject to copyright under 17 USC 105 and is also made available for use under a CC0 license.

Supplement I: Correlations between Manual and volBrain Segmentation

Neurotypical Controls

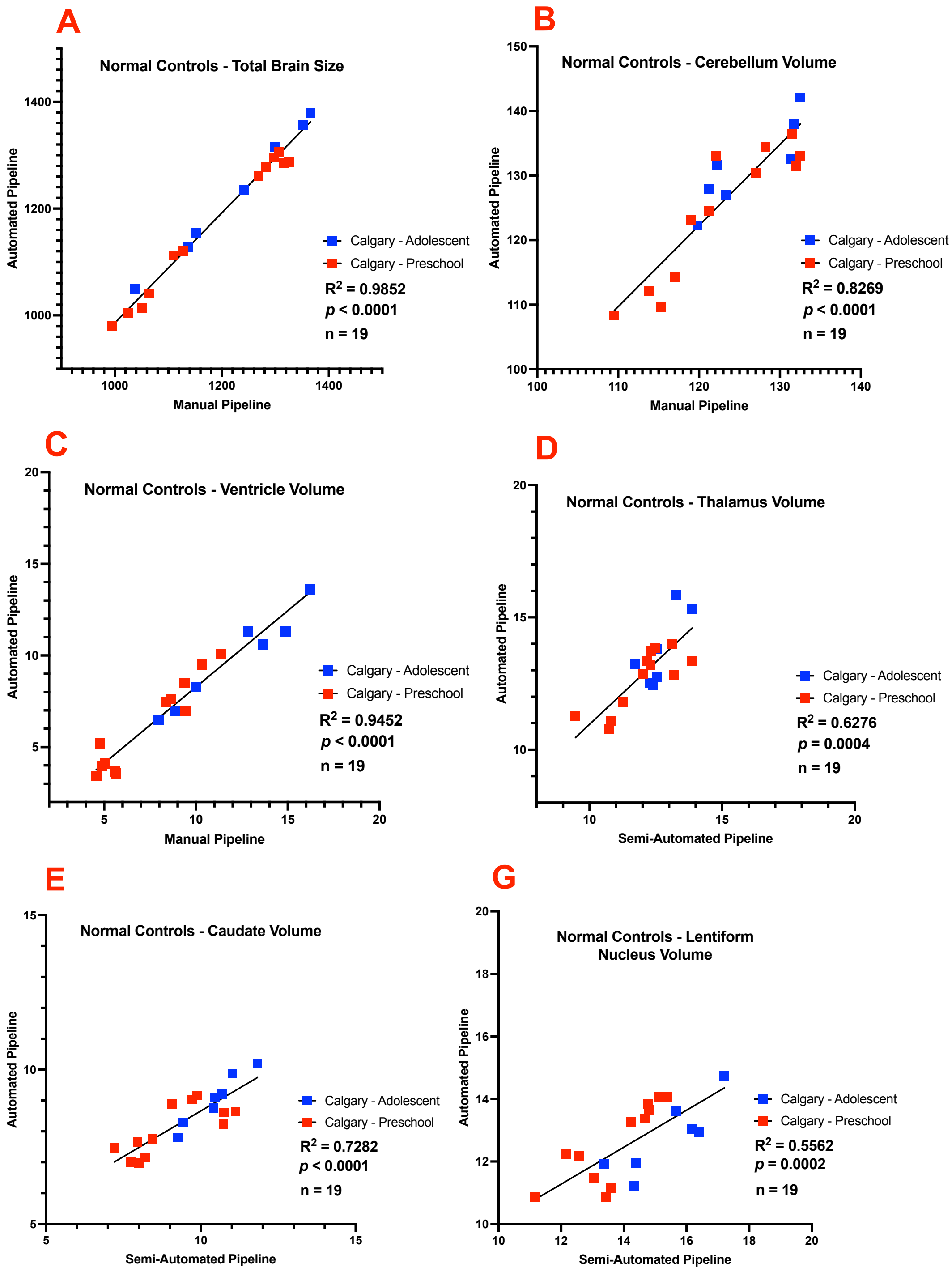

Figure I1. Correlations between volBrain's automated and the manual approach in neurotypical controls for the A.) Total brain volume B.) Cerebellum volume C.) Ventricle volume D.) Thalamic volume E.) Caduate volume and F.) Lentiform nucleus volume. Participants from the Calgary preschool MRI data set are shown in red and participants from the adolescent Calgary dataset are shown in blue. All volumes are shown in cm^3.

medRxiv preprint doi: https://doi.org/10.1101/2025.02.18.25322304; this version posted February 20, 2025. The copyright holder for this preprint
(which was not certified by peer review) is the author/funder, who has granted medRxiv a license to display the preprint in perpetuity.
This article is a US Government work. It is not subject to copyright under 17 USC 105 and is also made available
for use under a CC0 license.

Juvenile GM1 Patients

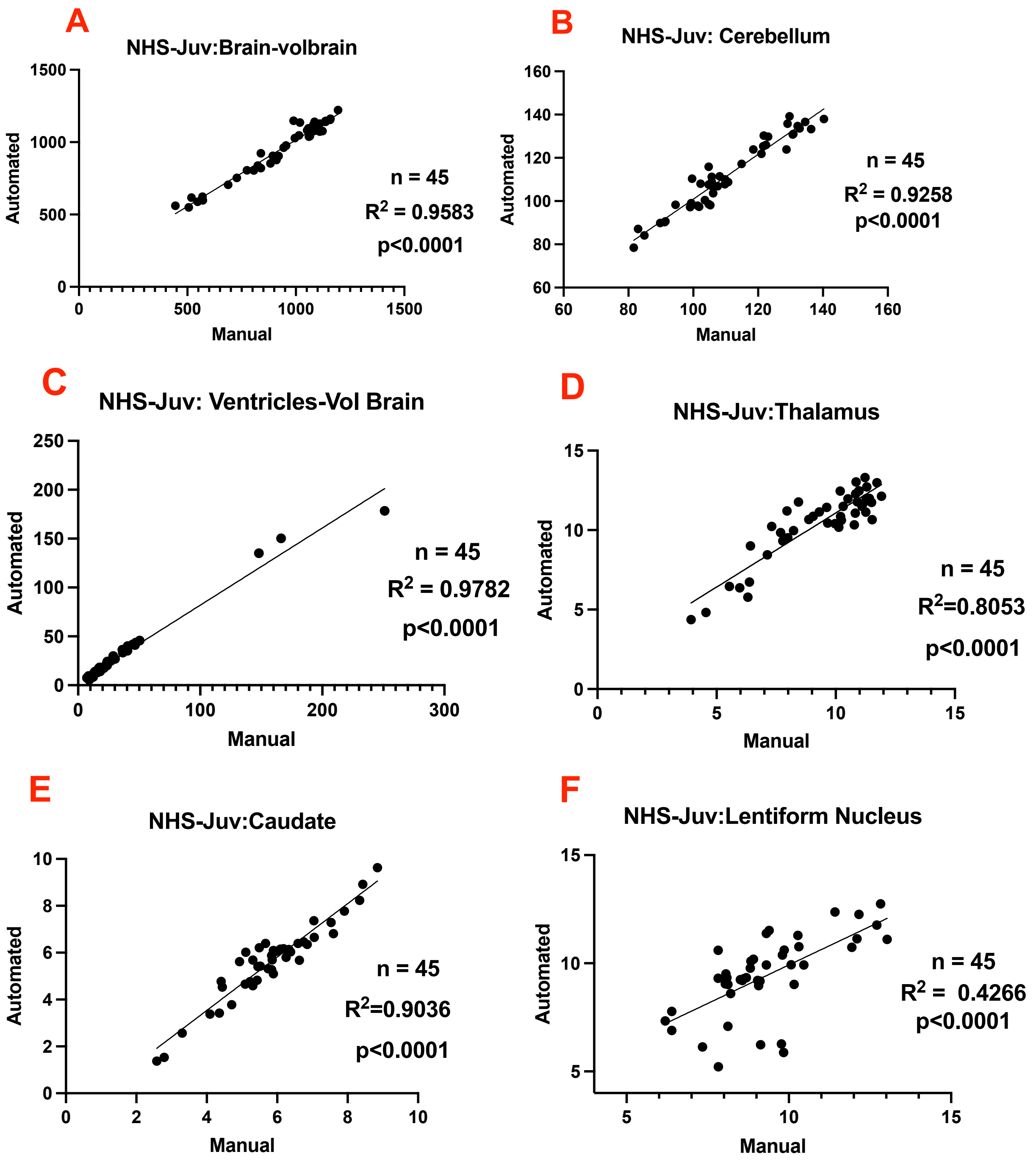

Figure I2. Correlations between volBrain's automated and the manual approach in natural history study (NHS) juvenile GM1 gangliosidosis patients for the A.) Total brain volume B.) Cerebellum volume C.) Ventricle volume D.) Thalamic volume E.) Caduate volume and F.) Lentiform nucleus volume. All volumes are shown in cm^3.

medRxiv preprint doi: https://doi.org/10.1101/2025.02.18.25322304; this version posted February 20, 2025. The copyright holder for this preprint
(which was not certified by peer review) is the author/funder, who has granted medRxiv a license to display the preprint in perpetuity.
This article is a US Government work. It is not subject to copyright under 17 USC 105 and is also made available
for use under a CC0 license.

Late-Infantile GM1 Patients

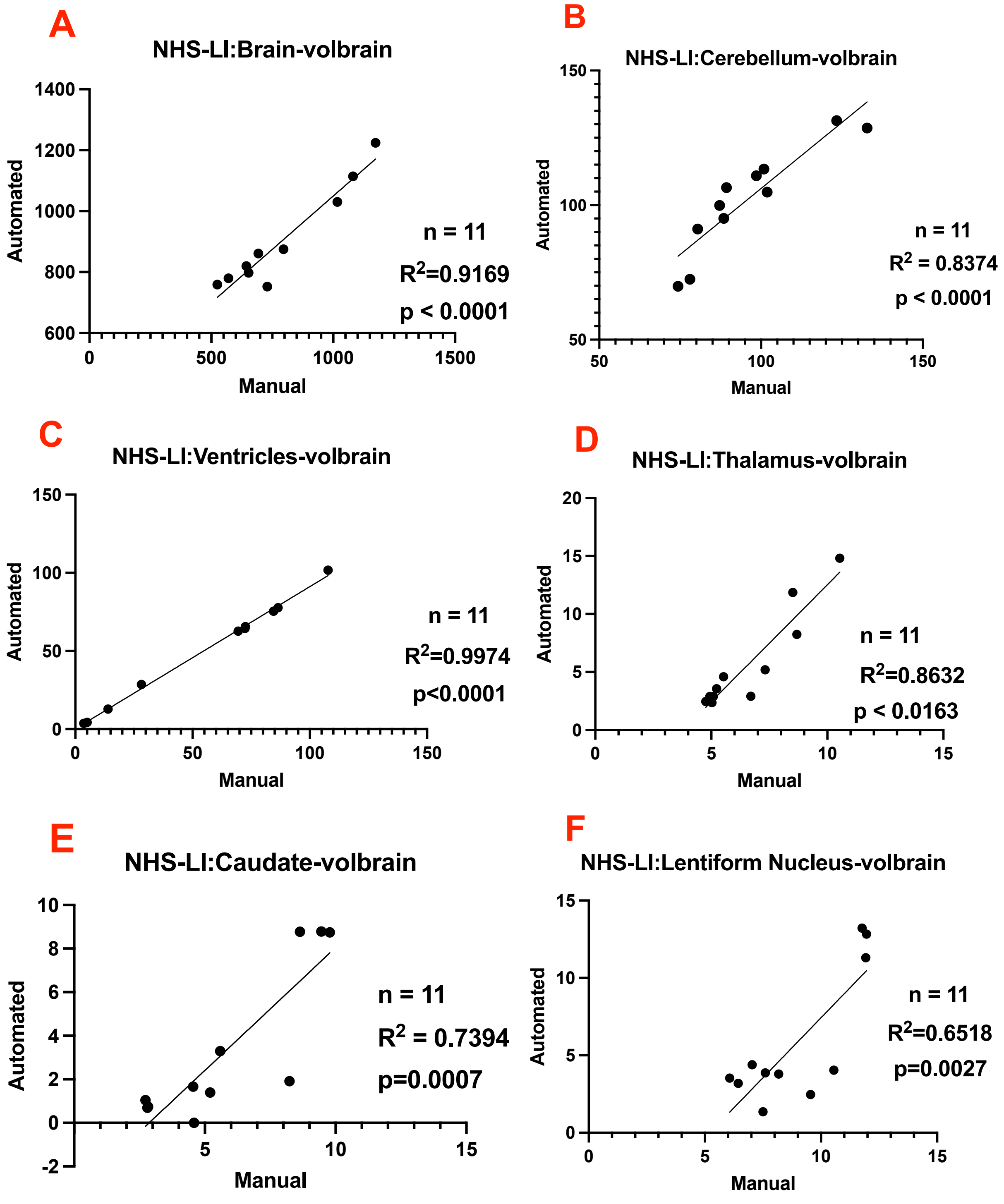

Figure I3. Correlations between volBrain's automated and the manual approach in natural history study (NHS) late-infantile GM1 gangliosidosis patients for the A.) Total brain volume B.) Cerebellum volume C.) Ventricle volume D.) Thalamic volume E.) Caduate volume and F.) Lentiform nucleus volume. All volumes are shown in cm^3.

medRxiv preprint doi: https://doi.org/10.1101/2025.02.18.25322304; this version posted February 20, 2025. The copyright holder for this preprint
(which was not certified by peer review) is the author/funder, who has granted medRxiv a license to display the preprint in perpetuity.
This article is a US Government work. It is not subject to copyright under 17 USC 105 and is also made available
for use under a CC0 license.

Supplement J: Correlations between Manual and FSL Segmentation

Neurotypical Controls

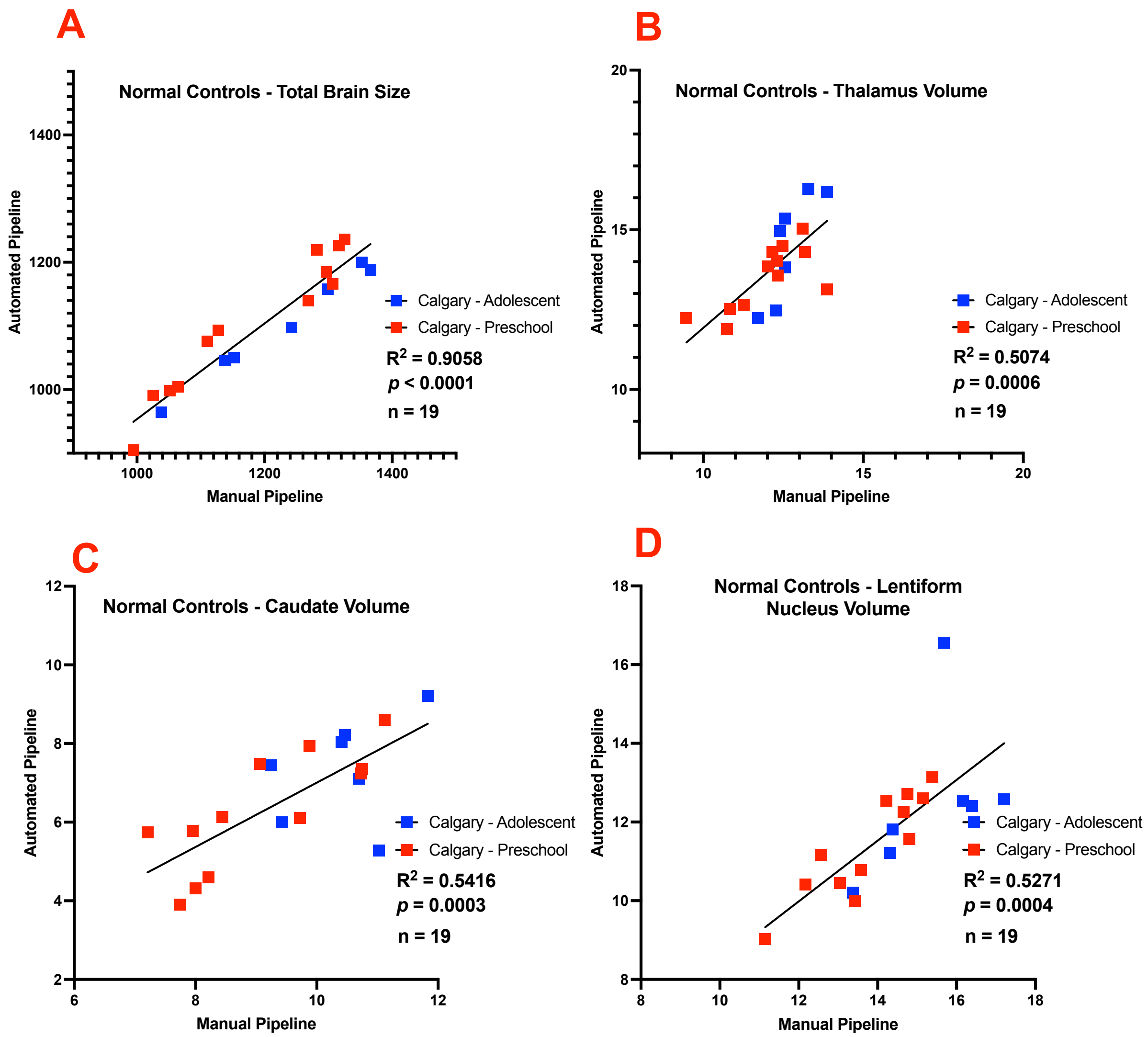

Figure J1. Correlations between FSL's automated and the manual approach in neurotypical controls for the A.) Total brain volume B.) Thalamic volume C.) Caduate volume and D.) Lentiform nucleus volume. Participants from the Calgary preschool MRI data set are shown in red and participants from the adolescent Calgary dataset are shown in blue. All volumes are shown in cm^3.

medRxiv preprint doi: https://doi.org/10.1101/2025.02.18.25322304; this version posted February 20, 2025. The copyright holder for this preprint (which was not certified by peer review) is the author/funder, who has granted medRxiv a license to display the preprint in perpetuity.
This article is a US Government work. It is not subject to copyright under 17 USC 105 and is also made available for use under a CC0 license.

Juvenile GM1 Patients

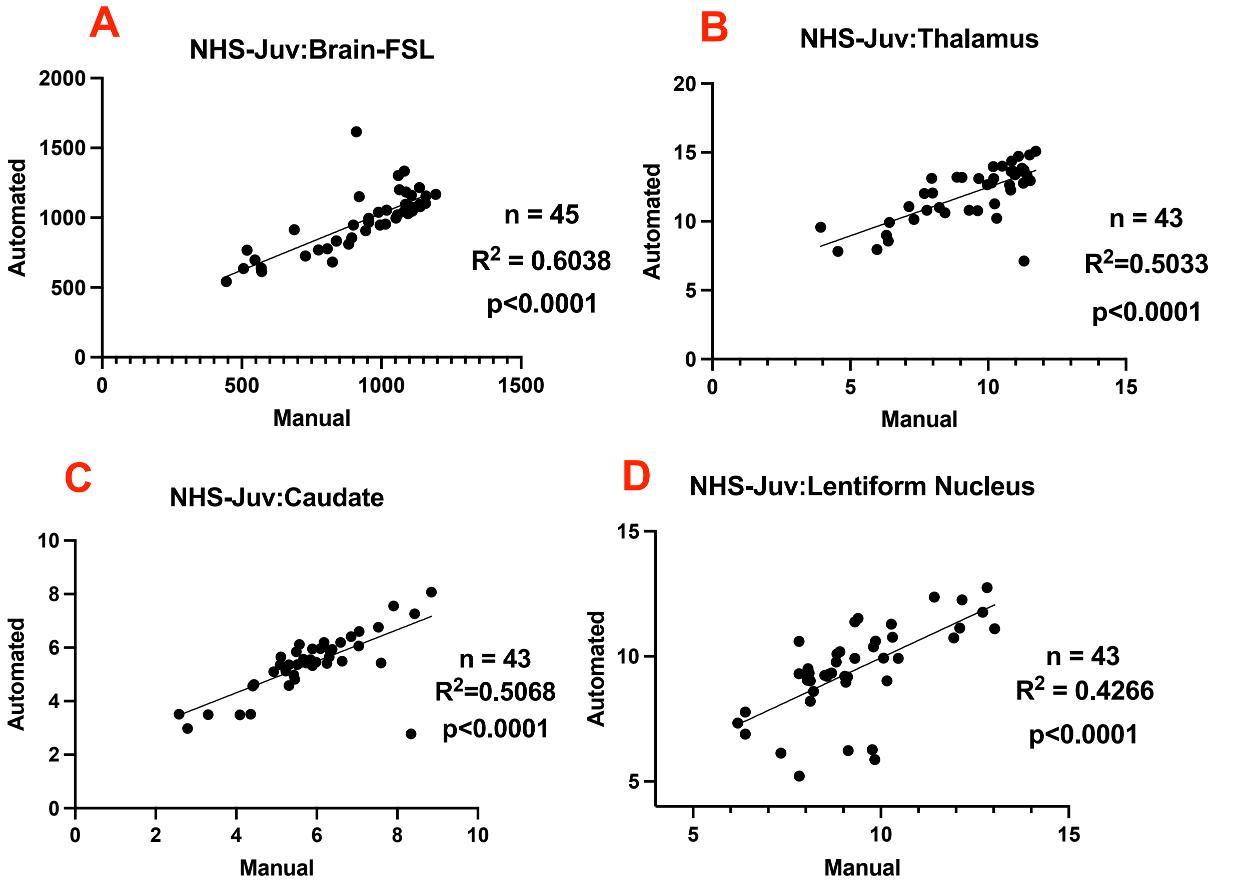

Figure J2. Correlations between FSL's automated and the manual approach in natural history study (NHS) juvenile GM1 gangliosidosis patients for the A.) Total brain volume B.) Thalamic volume C.) Caduate volume and D.) Lentiform nucleus volume. All volumes are shown in cm^3.

medRxiv preprint doi: https://doi.org/10.1101/2025.02.18.25322304; this version posted February 20, 2025. The copyright holder for this preprint (which was not certified by peer review) is the author/funder, who has granted medRxiv a license to display the preprint in perpetuity. This article is a US Government work. It is not subject to copyright under 17 USC 105 and is also made available for use under a CC0 license.

Late-Infantile GM1 Patients

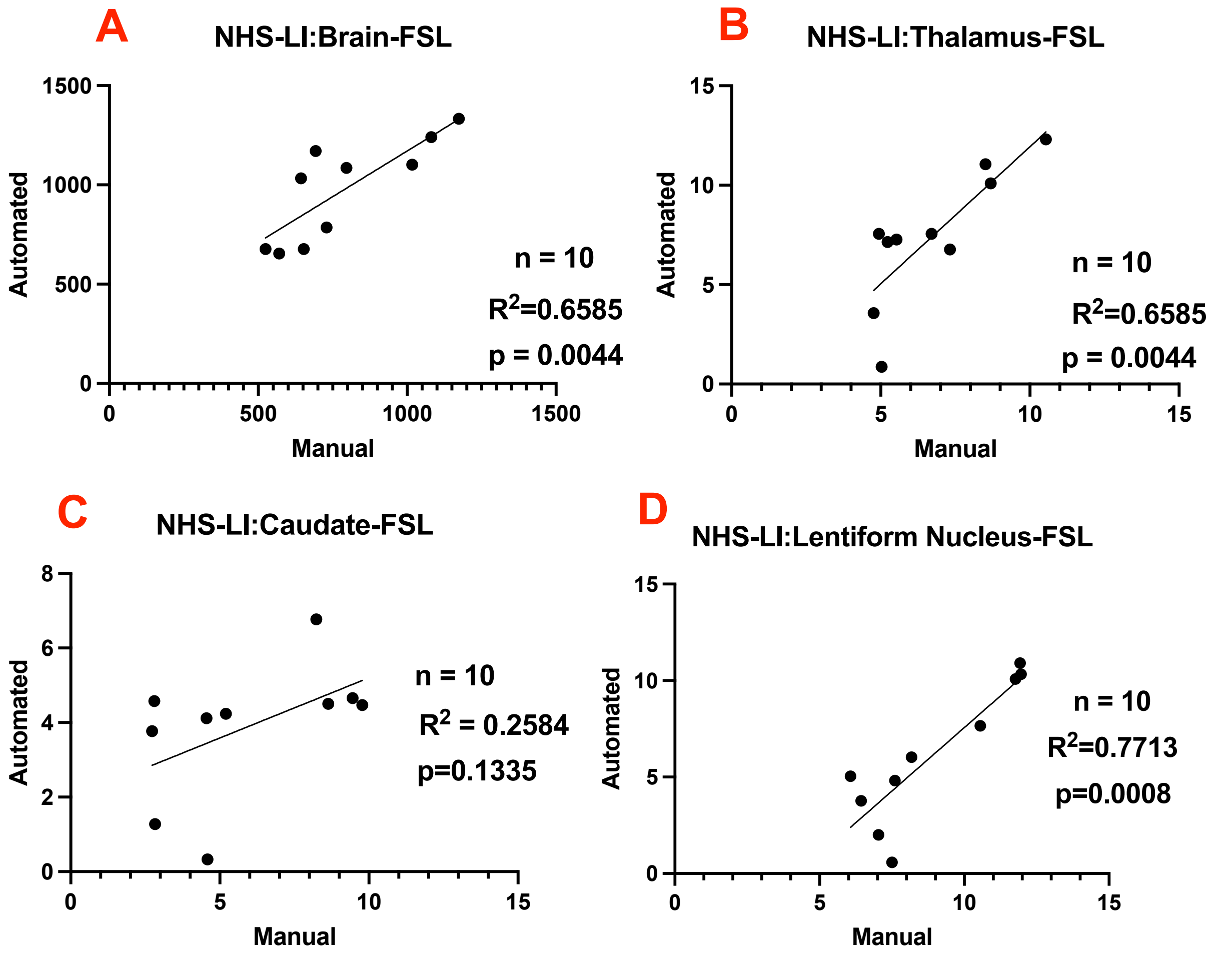

Figure J3. Correlations between FSL's automated and the manual approach in natural history study (NHS) juvenile GM1 gangliosidosis patients for the A.) Total brain volume B.) Thalamic volume C.) Caduate volume and D.) Lentiform nucleus volume. All volumes are shown in cm^3.

medRxiv preprint doi: https://doi.org/10.1101/2025.02.18.25322304; this version posted February 20, 2025. The copyright holder for this preprint (which was not certified by peer review) is the author/funder, who has granted medRxiv a license to display the preprint in perpetuity. This article is a US Government work. It is not subject to copyright under 17 USC 105 and is also made available for use under a CC0 license.

Supplement K: Correlations between Manual and SPM Segmentation

Neurotypical Controls

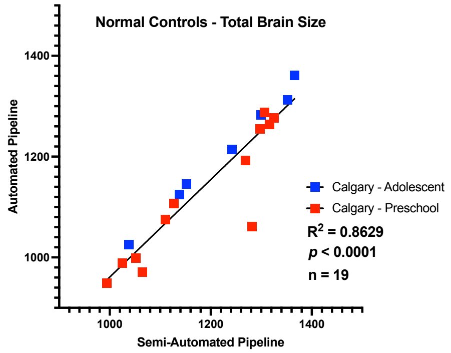

Figure K1. Correlations between SPM's automated and the manual approach in neurotypical controls for the total brain volume. Participants from the Calgary preschool MRI data set are shown in red and participants from the adolescent Calgary dataset are shown in blue. All volumes are shown in cm^3.

Juvenile GM1 Patients

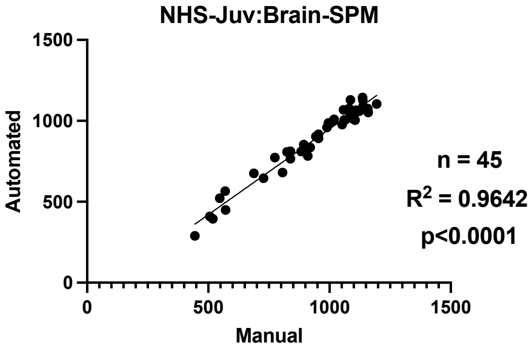

Figure K2. Correlations between SPM's automated and the manual approach in natural history study (NHS) juvenile GM1 gangliosidosis patients for total brain volume. All volumes are shown in cm^3.

medRxiv preprint doi: https://doi.org/10.1101/2025.02.18.25322304; this version posted February 20, 2025. The copyright holder for this preprint (which was not certified by peer review) is the author/funder, who has granted medRxiv a license to display the preprint in perpetuity.
This article is a US Government work. It is not subject to copyright under 17 USC 105 and is also made available for use under a CC0 license.

Late-Infantile GM1 Patients

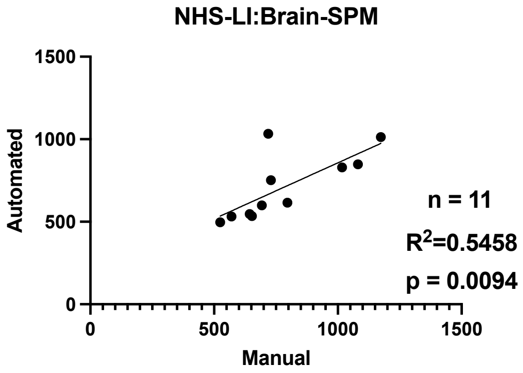

Figure K3. Correlations between SPM's automated and the manual approach in natural history study (NHS) late-infantile GM1 gangliosidosis patients for total brain volume. All volumes are shown in cm^3.

medRxiv preprint doi: https://doi.org/10.1101/2025.02.18.25322304; this version posted February 20, 2025. The copyright holder for this preprint (which was not certified by peer review) is the author/funder, who has granted medRxiv a license to display the preprint in perpetuity.
This article is a US Government work. It is not subject to copyright under 17 USC 105 and is also made available for use under a CC0 license.

Supplement L: Correlations between Manual and Headreco Segmentation

Neurotypical Controls

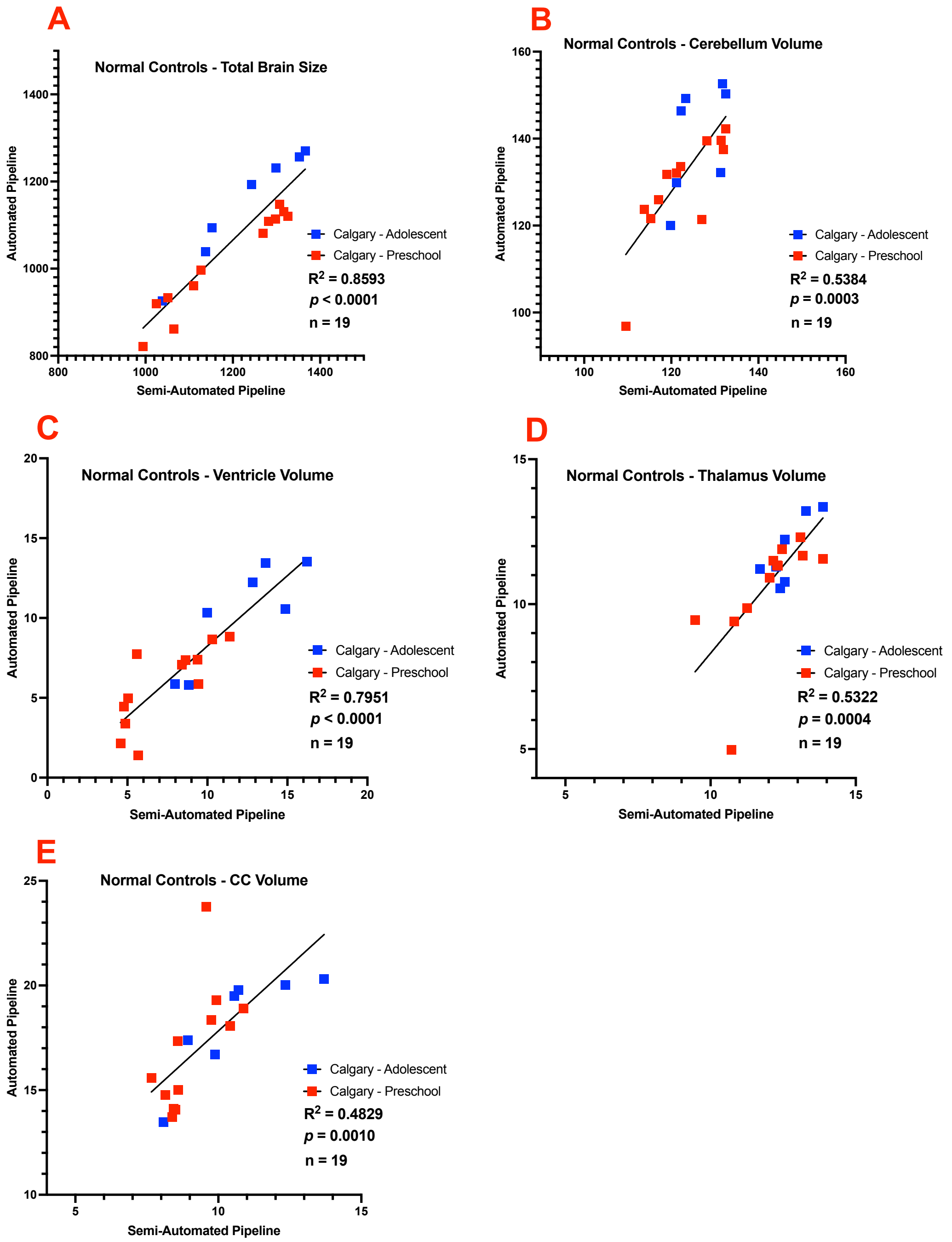

Figure L1. Correlations between Headreco's automated and the manual approach in neurotypical controls for the A.) Total brain volume B.) Cerebellum volume C.) Ventricle volume D.) Thalamic volume and E.) Corpus callosum volume. Participants from the Calgary preschool MRI data set are shown in red and participants from the adolescent Calgary dataset are shown in blue. All volumes are shown in cm^3.

medRxiv preprint doi: https://doi.org/10.1101/2025.02.18.25322304; this version posted February 20, 2025. The copyright holder for this preprint (which was not certified by peer review) is the author/funder, who has granted medRxiv a license to display the preprint in perpetuity. This article is a US Government work. It is not subject to copyright under 17 USC 105 and is also made available for use under a CC0 license.

Juvenile GM1 Patients

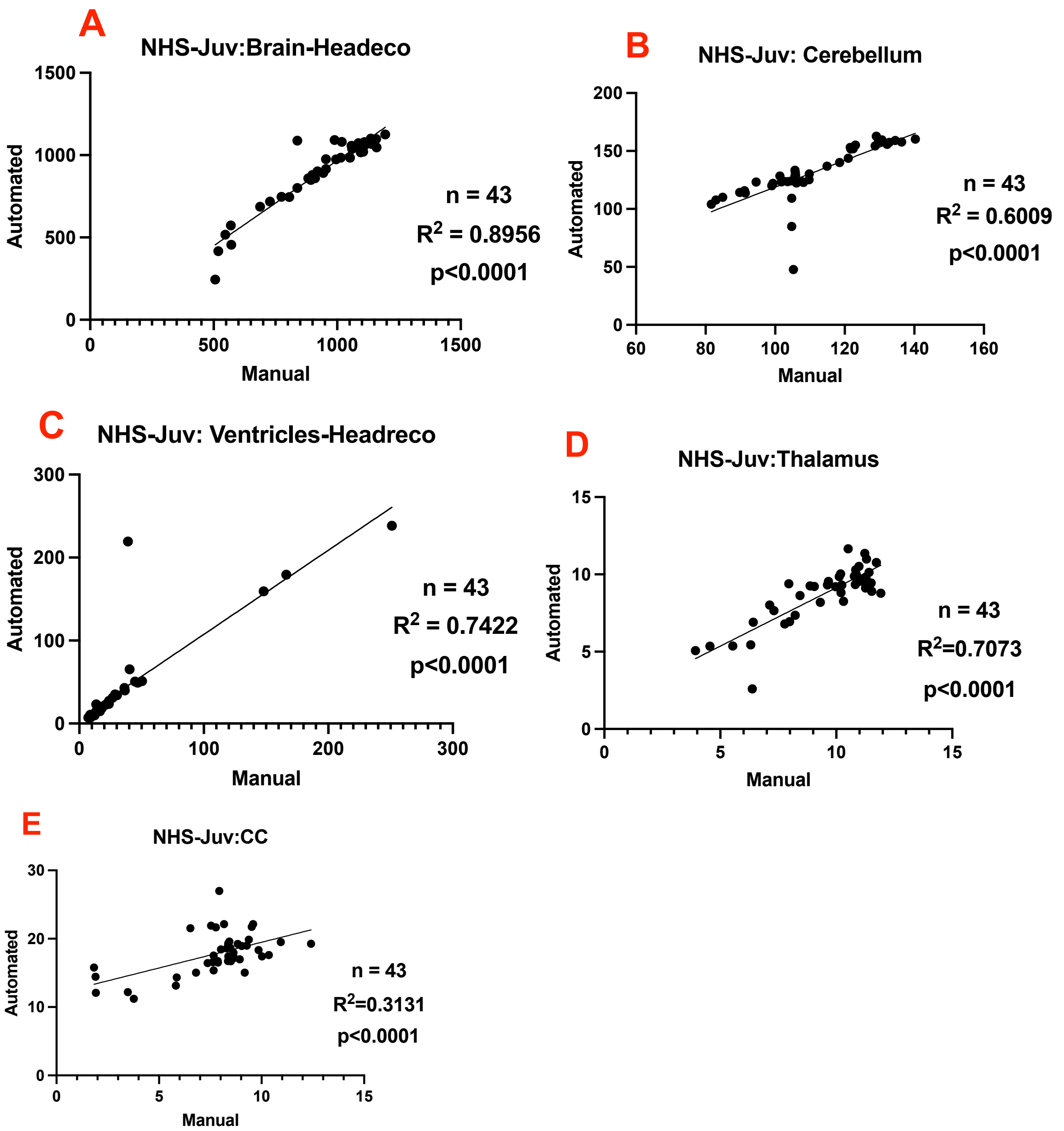

Figure L2. Correlations between Headreco's automated and the manual approach in natural history study (NHS) juvenile GM1 gangliosidosis patietns for the A.) Total brain volume B.) Cerebellum volume C.) Ventricle volume D.) Thalamic volume and E.) Corpus callosum volume. All volumes are shown in cm^3.

medRxiv preprint doi: https://doi.org/10.1101/2025.02.18.25322304; this version posted February 20, 2025. The copyright holder for this preprint
(which was not certified by peer review) is the author/funder, who has granted medRxiv a license to display the preprint in perpetuity.
This article is a US Government work. It is not subject to copyright under 17 USC 105 and is also made available
for use under a CC0 license.

Late-Infantile GM1 Patients

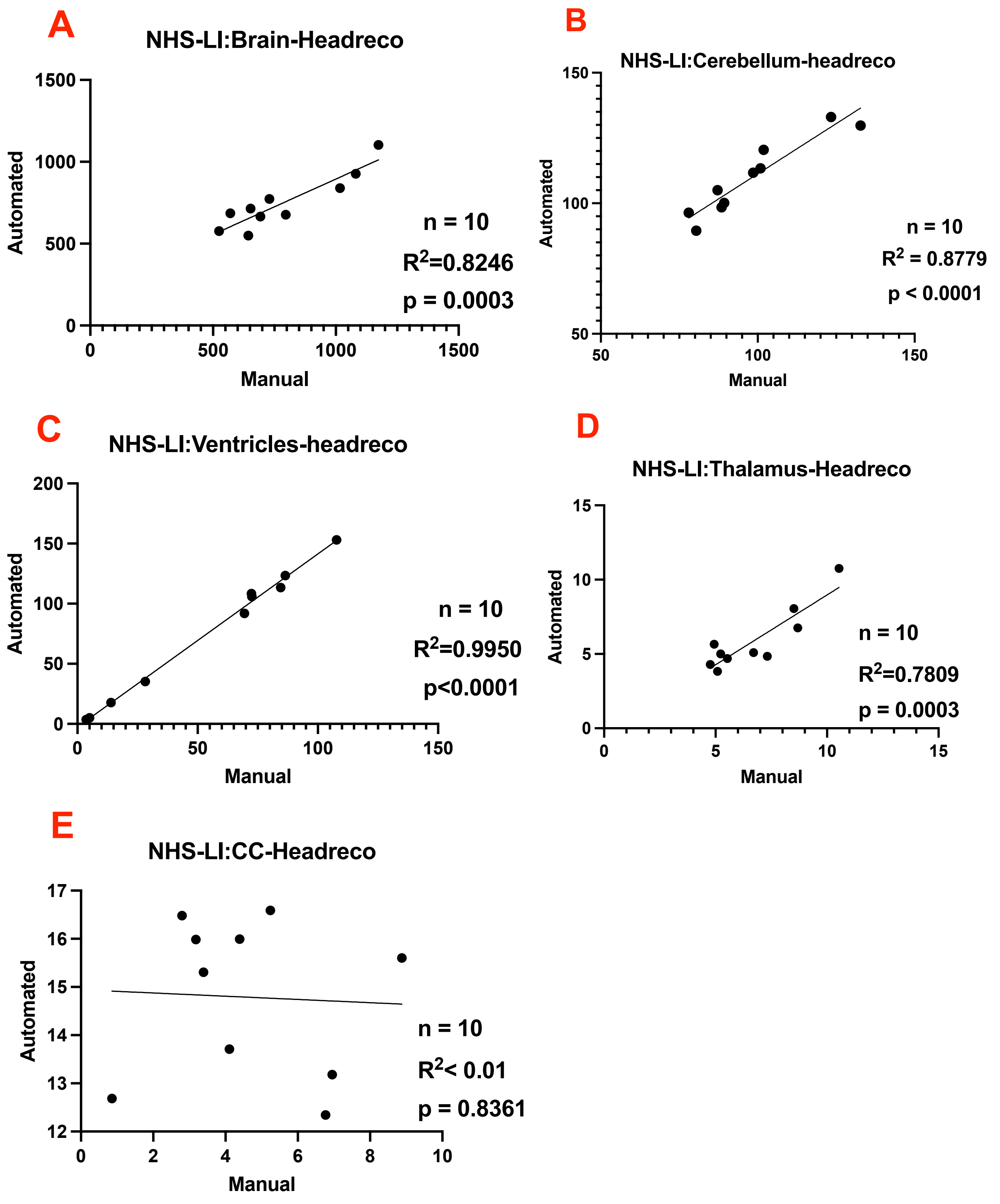

Figure L3. Correlations between Headreco's automated and the manual approach in natural history study (NHS) late-infantile GM1 gangliosidosis patietns for the A.) Total brain volume B.) Cerebellum volume C.) Ventricle volume D.) Thalamic volume and E.) Corpus callosum volume. All volumes are shown in cm^3.

medRxiv preprint doi: https://doi.org/10.1101/2025.02.18.25322304; this version posted February 20, 2025. The copyright holder for this preprint (which was not certified by peer review) is the author/funder, who has granted medRxiv a license to display the preprint in perpetuity.
This article is a US Government work. It is not subject to copyright under 17 USC 105 and is also made available for use under a CC0 license.

Supplement M: Slopes and Intercepts between Manual and Automated Segmentation

Table MI. Comparison of slopes and intercepts of the linear regression modeling between the 5 fully automated segmentation pipelines and the manual pipeline in the 7 different brain regions for the neurotypical controls. N/A are designations where the region was not calculated using the specified segmentation algorithm. The slope of the linear regression line is given in the top row, and the intercept is given in the bottom row for each cell. The 95% confidence interval for the slope and intercept shown in the brackets. For all linear regression modeling the automated volumetric calculations were along the y-axis, and the manual volumetric calculations were along the x-axis.

Structure	Freesurfer	FSL	volBrain	SPM	SimNIBS (SPM+CAT)
Whole Brain	0.99 [0.76, 1.23]<br>-99.32 [-380.9, 182.3]	0.75 [0.63, 0.88]<br>202.1 [52.83, 351.5]	1.03 [0.97, 1.10]<br>-44.99 [-122.8, 32.82]	0.97 [0.77, 1.16]<br>-6.21 [-243.7, 231.2]	0.98 [0.78, 1.19]<br>112.6 [-357.4, 132.2]
Ventricles	0.68 [0.40, 0.95]<br>2.77 [0.13, 5.41]	N/A	0.83 [0.73, 0.94]<br>-0.05 [-1.04, 0.95]	N/A	0.88 [0.65, 1.11]<br>-0.58 [-2.81, 1.64]
Cerebellum	1.27 [0.84, 1.71]<br>25.61 [-80.32, 27.75]	N/A	1.26 [0.96, 1.55]<br>-28.53 [-65.00, 7.93]	N/A	1.38 [0.73, 2.04]<br>-38.04 [-119.2, 43.11]
Thalamus	1.15 [0.60, 1.70]<br>0.34 [-6.39, 7.08]	0.87 [0.43, 1.30]<br>3.26 [-2.11, 8.62]	0.94 [0.57, 1.31]<br>1.55 [-3.00, 6.10]	N/A	1.21 [0.63,1.78]<br>-3.75 [-10.85, 3.35]
Caudate	0.89 [0.67, 1.10]<br>-0.39 [-2.47, 1.68]	0.82 [0.43, 1.20]<br>-1.18 [-4.90, 2.55]	0.59 [0.41, 0.78]<br>2.75 [0.96, 4.53]	N/A	N/A
Lentiform Nucleus	0.80 [0.54, 1.05]<br>2.55 [-1.06, 6.16]	0.77 [0.40, 1.15]<br>0.72 [-4.67, 6.12]	0.59 [0.32, 0.86]<br>2.13 [0.28, 8.08]	N/A	N/A
Corpus Callosum	0.09 [-0.08, 0.27]<br>2.04 [0.33, 3.74]	N/A	N/A	N/A	1.25 [0.59, 1.90]<br>5.38 [-1.05, 11.81]

Table MII. Comparison of slopes and intercepts of the linear regression modeling between the 5 fully automated segmentation pipelines and the manual pipeline in the 7 different brain regions for the juvenile GM1 patients. N/A are designations where the region was not calculated using the specified segmentation algorithm. The slope of the linear regression line is given in the top row, and the intercept is given in the bottom row for each cell. The 95% confidence interval for the slope and intercept shown in the brackets. For all linear regression modeling the automated volumetric calculations were along the y-axis, and the manual volumetric calculations were along the x-axis.

Structure	Freesurfer	FSL	volBrain	SPM	SimNIBS (SPM+CAT)
Whole Brain	0.85 [0.77, 0.95]<br>74.26 [-10.34, 158.9]	0.82 [0.63, 1.02]<br>215.7 [20.80, 410.5]	0.92 [0.86, 0.98]<br>92.83 [36.75, 148.9]	1.06 [1.00, 1.12]<br>-109.9 [-170.1, -46.67]	1.04 [0.92, 1.15]<br>-68.93 [-177.2, 39.38]
Ventricles	0.97 [0.92, 1.02]<br>0.80 [-0.40, 2.00]	N/A	0.79 [0.75, 0.83]<br>2.89 [0.88, 4.91]	N/A	0.91 [0.87, 0.95]<br>0.22 [-2.24, 2.68]
Cerebellum	1.00 [0.90, 1.11]<br>5.48 [-6.27, 17.24]	N/A	1.04 [0.95, 1.12]<br>-2.67 [-12.42, 7.09]	N/A	1.15 [0.86, 1.45]<br>3.55 [-29.67, 36.78]
Thalamus	0.63 [0.39, 0.88]<br>6.53 [4.09, 8.98]	0.71 [0.48, 0.93]<br>5.42 [3.30, 7.55]	0.94 [0.79, 1.08]<br>1.74 [0.38, 3.11]	N/A	0.76 [0.60, 0.91]<br>1.58 [0.09, 3.07]
Caudate	0.83 [0.70, 0.96]<br>0.54 [-0.29, 1.37]	0.59 [0.41, 0.77]<br>1.96 [0.86, 3.06]	1.14 [1.02, 1.26]<br>-1.02 [-1.72, -0.33]	N/A	N/A
Lentiform Nucleus	0.97 [0.69, 1.25]<br>0.39 [-2.28, 3.06]	0.70 [0.44, 0.96]<br>2.94 [0.51, 5.36]	0.70 [0.44, 0.96]<br>2.99 [0.51, 5.46]	N/A	N/A
Corpus Callosum	0.23 [0.14, 0.32]<br>0.79 [0.04, 1.54]	N/A	N/A	N/A	0.75 [0.40, 1.10]<br>11.96 [9.11, 14.81]

medRxiv preprint doi: https://doi.org/10.1101/2025.02.18.25322304; this version posted February 20, 2025. The copyright holder for this preprint (which was not certified by peer review) is the author/funder, who has granted medRxiv a license to display the preprint in perpetuity.
This article is a US Government work. It is not subject to copyright under 17 USC 105 and is also made available for use under a CC0 license.

Table MIII. Comparison of slopes and intercepts of the linear regression modeling between the 5 fully automated segmentation pipelines and the manual pipeline in the 7 different brain regions for the late-infantile GM1 patients. N/A are designations where the region was not calculated using the specified segmentation algorithm. The slope of the linear regression line is given in the top row, and the intercept is given in the bottom row for each cell. The 95% confidence interval for the slope and intercept shown in the brackets. For all linear regression modeling the automated volumetric calculations were along the y-axis, and the manual volumetric calculations were along the x-axis.

Structure	Freesurfer	FSL	volBrain	SPM	SimNIBS (SPM+CAT)
Whole Brain	0.32 [-0.04, 00.69]<br>657.2 [361.0, 953.4]	0.68 [-1.21, 2.57]<br>593.0 [-934.2, 2120]	0.70 [0.53, 0.87]<br>349.7 [209.4, 490.0]	0.68 [0.21, 1.15]<br>178.6 [-198.5, 555.8]	0.68 [0.42, 0.93]<br>217.8 [10.00, 425.6]
Ventricles	0.68 [0.35, 1.00]<br>7.70 [-13.27, 28.62]	N/A	0.91 [0.87, 0.95]<br>0.22 [-2.24, 2.68]	N/A	1.44 [1.36, 1.52]<br>-2.55 [-7.98, 2.87]
Cerebellum	0.63 [0.27, 0.99]<br>38.87 [3.25, 74.49]	N/A	0.98 [0.66, 1.31]<br>-8.23 [-23.71, 39.85]	N/A	0.85 [0.61, 1.09]<br>25.10 [1.70, 48.49]
Thalamus	0.97 [0.23, 1.71]<br>3.51 [-1.65, 8.66]	1.38 [0.57, 2.19]<br>-1.85 [-7.50, 3.80]	2.02 [1.41, 2.62]<br>-7.63 [-11.77, -3.50]	N/A	0.92 [0.55, 1.28]<br>-0.23 [-2.73, 2.28]
Caudate	0.70 [0.47, 0.94]<br>0.73 [-2.47, 2.27]	0.32 [-0.12, 0.77]<br>1.97 [-0.92, 4.86]	1.13 [0.62, 1.63]<br>-3.24 [-6.47, 0.00]	N/A	N/A
Lentiform Nucleus	1.22 [0.51, 1.94]<br>-3.26 [-9.94, 3.43]	1.32 [0.73, 1.90]<br>-5.59 [-10.95, -0.22]	1.55 [0.70, 2.41]<br>-8.08 [-15.96, -0.20]	N/A	N/A
Corpus Callosum	0.06 [-0.14, 0.26]<br>1.64 [0.60, 2.69]	N/A	N/A	N/A	-0.05 [-0.55, 0.45]<br>15.03 [12.56, 17.51]

medRxiv preprint doi: https://doi.org/10.1101/2025.02.18.25322304; this version posted February 20, 2025. The copyright holder for this preprint (which was not certified by peer review) is the author/funder, who has granted medRxiv a license to display the preprint in perpetuity. This article is a US Government work. It is not subject to copyright under 17 USC 105 and is also made available for use under a CC0 license.

Supplementary References

1. National Human Genome Research Institute. Natural History of Glycosphingolipid Storage Disorders and Glycoprotein Disorders ClinicalTrials.gov identifier: NCT00029965. Updated August 7, 2024. Accessed August 12, 2024. https://clinicaltrials.gov/study/NCT00029965.
2. Reynolds JE, Long X, Paniukov D, Bagshawe M, Lebel C. Calgary Preschool magnetic resonance imaging (MRI) dataset. Data Brief. 2020 Jan 31;29:105224. doi: 10.1016/j.dib.2020.105224. PMID: 32071993; PMCID: PMC7016255.
3. Mah A, Geeraert B, Lebel C (2017) Detailing neuroanatomical development in late childhood and early adolescence using NODDI. PLoS ONE 12(8): e0182340. https://doi.org/10.1371/journal.pone.0182340.
